# High-Throughput Discovery of Chloroplast and Mitochondrial DNA Polymorphisms in Brassicaceae Species by ORG-EcoTILLING

**DOI:** 10.1371/journal.pone.0047284

**Published:** 2012-11-21

**Authors:** Chang-Li Zeng, Guang-Yong Wang, Jian-Bo Wang, Gui-Xin Yan, Bi-Yun Chen, Kun Xu, Jun Li, Gui-Zhen Gao, Xiao-Ming Wu, Bo Zhao, Lei Liu

**Affiliations:** 1 Oil Crop Research Institute, Chinese Academy of Agricultural Sciences, Key Laboratory of Biology and Genetic Improvement of Oil Crops, Ministry of Agriculture, Wuhan, China; 2 College of Life Sciences, Jianghan University, Wuhan, China; 3 Key Laboratory of MOE for Plant Developmental Biology, College of Life Sciences, Wuhan University, Wuhan, China; University of Hong Kong, China

## Abstract

**Background:**

Information on polymorphic DNA in organelle genomes is essential for evolutionary and ecological studies. However, it is challenging to perform high-throughput investigations of chloroplast and mitochondrial DNA polymorphisms. In recent years, EcoTILLING stands out as one of the most universal, low-cost, and high-throughput reverse genetic methods, and the identification of natural genetic variants can provide much information about gene function, association mapping and linkage disequilibrium analysis and species evolution. Until now, no report exists on whether this method is applicable to organelle genomes and to what extent it can be used.

**Methodology/Principal Findings:**

To address this problem, we adapted the CEL I-based heteroduplex cleavage strategy used in Targeting Induced Local Lesions in Genomes (TILLING) for the discovery of nucleotide polymorphisms in organelle genomes. To assess the applicability and accuracy of this technology, designated ORG-EcoTILLING, at different taxonomic levels, we sampled two sets of taxa representing accessions from the Brassicaceae with three chloroplast genes (*accD*, *matK* and *rbcL*) and one mitochondrial gene (*atp6*). The method successfully detected nine, six and one mutation sites in the *accD*, *matK* and *rbcL* genes, respectively, in 96 *Brassica* accessions. These mutations were confirmed by DNA sequencing, with 100% accuracy at both inter- and intraspecific levels. We also detected 44 putative mutations in *accD* in 91 accessions from 45 species and 29 genera of seven tribes. Compared with DNA sequencing results, the false negative rate was 36%. However, 17 SNPs detected in *atp6* were completely identical to the sequencing results.

**Conclusions/Significance:**

These results suggest that ORG-EcoTILLING is a powerful and cost-effective alternative method for high-throughput genome-wide assessment of inter- and intraspecific chloroplast and mitochondrial DNA polymorphisms. It will play an important role in evolutionary and ecological biology studies, in identification of related genes associated with agronomic importance such as high yield and improved cytoplasmic quality, and for identifying mitochondrial point mutations responsible for diseases in humans and other animals.

## Introduction

The fields of ecology and phylogeny are currently experiencing a renaissance spurred by the rapid development of molecular detection techniques. By detecting genetic variation in both nuclear and organelle genomes, molecular markers have made a profound and significant contribution to studies of evolution, domestication, speciation, evolution of genomes, genetic diversity, population structure, levels of gene flow, patterns of historical biogeography and analyses of parentage assignments, genetic variability and inbreeding [1]–[6]. Mitochondria and chloroplasts are important organelles in eukaryotic organisms, and both genomes contain two vital sets of genes [7]. Plant mitochondrial (mt) DNAs are extremely variable in size (200–2400 kb) [8], [9], whereas animal mtDNAs are essentially invariant in gene order among all vertebrates [10]. Chloroplasts (cps) contain their own small genomes, which averages 120 to 200 kilobase pairs (kb) among almost all chloroplast-containing organisms [11]. The plant chloroplast genome shares many features with animal mtDNA and the two have been referred to as ‘natural counterparts’ [12]. In animals, mtDNA is characterized by a small size, high copy number, relatively conserved gene order, ready availability of primers and rapid substitution rates [13], whereas in plants, the chloroplast genome is associated with a conserved gene order, widespread availability of primers and a general lack of heteroplasmy and recombination [14]. As a result, the nonrecombinant, uniparentally inherited and effectively haploid nature of chloroplast and mitochondrial genomes makes them useful tools for studies on plant and animal evolution, respectively [14]. Chloroplast genomes are predominantly maternally inherited (mainly transmitted through the embryos of seeds) and so can reveal maternal lineages [15]–[18], enabling divergent patterns of variation to be detected in these genomes compared with those revealed by nuclear markers. For example, investigation of chloroplast genomes can be used to document the maternal parent of hybrid plants [19], define organelle haplotypes [20] or detect introgression [21], [22].

In order to detect informative polymorphisms for phylogenetic studies, a large number of molecular technologies have been developed for assessing chloroplast and mitochondrial genomes. Conventional methods include DNA restriction mapping [23], [24], the use of hybridization-based restriction fragment length polymorphisms (RFLP) [25], PCR-RFLP [26]–[28] and nucleotide sequence analysis [29], [30]. However, each of these methods have some disadvantages, including low resolution, labor intensity and the requirement for a large amount of isolated DNA. For instance, although Chloroplast microsatellites (cpSSR) have proven to be useful markers for gaining insights into the genetic relationships of closely related species and populations [31], [14], they are limited to study of closely related taxa where there are no or few nucleotide substitutions, especially in the coding regions of the chloroplast genome [32].

Because single-base substitutions and small insertions and deletions (INDELs) are the most common forms of genetic variation in organelle genomes, nucleotide sequence analysis of specific chloroplast and mitochondrial genes has been widely used in phylogenetic and ecological studies. Several key genes, such as chloroplast *rbcL*, *matK* and *accD*, mitochondrial *atp*6 and a mitochondrial fragment COX1 play important roles in clarifying phylogenetic relationships amongst plants [33]–[37]. However, *rbcL*, *matK* and *accD* only represent about 2.9% of the genome. Next-generation sequencing technologies, such as Roche/454, Illumina/Solexa and Life/APG are able to generate large volumes of sequence data at relatively low cost [38], enabling complete coverage of organelle genomes. However, at present this is only feasible for relatively low level sampling of individuals [39], [40]. This has led to a debate over the relative worth of taxon sampling (investigating many lineages) compared with site sampling (investigating many sites from many genes from a few crucial lineages) in building phylogenetic trees from sequence data [41], [42]. Thus there is a need for new methods that have the features of high resolution, high throughput, and ease of use, accuracy and cost effectiveness.

TILLING (Targeting Induced Local Lesions in Genomes) and EcoTILLING were developed as a means for high-throughput discovery of DNA polymorphisms in nuclear genomes in EMS-mutagenized [43]–[47] and natural populations [48]. The initial development of these methods employed the mismatch-specific endonuclease CEL I to discover point mutations in genes, in conjunction with PCR-based screening [49], [50]. Subsequently there have been modifications of the approach using other detection methods, including the use of new generation sequencing coupled with multidimensional pooling for the identification of rare alleles in populations of rice and wheat [51].

Given the ability of CEL1 and a PCR-based strategy to detect single nucleotide polymorphisms (SNPs), we decided to explore whether this approach could be applied to organelle genomes. We therefore developed a method, designated “ORG- EcoTILLING”, for high-resolution, high-throughput detection of multiple polymorphisms in chloroplast and mitochondrial genes.

In order to assess the applicability and accuracy of ORG-EcoTILLING we sampled two sets of plant taxa representing different levels of variation within the Brassicaceae. The Brassicaceae include approximately 340 genera and 3350 species [52], of which the model plant *Arabidopsis thaliana* and domesticated species such as oilseed rape, Chinese cabbage, broccoli, turnip, radish, mustard, and other *Brassica* crops, are perhaps the most familiar [53]. Plants within the Brassicaceae have therefore been regarded as ideal materials for the study of evolutionary relationships [54], [55]. This study aimed to (1) develop a strategy for ORG-EcoTILLING to discriminate genetic SNPs and INDELs in chloroplast and mitochondrial genes of numerous Brassicaceae plant accessions; (2) assess the accuracy of ORG-EcoTILLING for the identification of cytoplasmic DNA Polymorphism; and (3) provide an effective, low-cost, high-throughput technology for identification of genes associated with agronomic importance such as high yield and improved cytoplasmic efficiency, analyzing phylogenetic relationships in plants, and identifying point mutations of mitochondrial genes responsible for diseases in humans and other animals.

## Materials and Methods

### Plant material

To evaluate the applicability and accuracy of ORG-EcoTILLING for detecting DNA variation in chloroplast genes at different taxonomic levels, two sets of plant specimens were sampled. The first set, composed of 91 representative accessions from 45 species and 29 genera of seven tribes (Trib. Brassiceae Hayek, Trib. Sisymbrieae DC, Trib. Matthioleae O.E. Schulz, Trib. Alysseae Gren. Et Godr, Trib. Arabideae DC, Trib. Hesperideae Prantl, and Trib. Lepidieae DC) (Table 1) was used to assess the applicability and accuracy at intertribal, intergeneric and interspecific levels. The second set sampled 90 accessions of cultivars of *Brassica napus*, and three accessions each of *B. rapa* and *B. oleracea* (Table 2), which were used to evaluate the applicability and accuracy of this method at intraspecific and interspecific levels.

**Table 1:** 91 accessions from Cruciferae used for ORG- EcoTILLING.

Sample code	Materials name	Origin	Trib	Genus	Species
A1	Changyouxiaoheiyoucai	China	Trib. Brassiceae Hayek	*Brassica*	*B. rapa*
A2	Xishui	China	Trib. Brassiceae Hayek	*Brassica*	*B. rapa*
A3	Guangfuqing	China	Trib. Brassiceae Hayek	*Brassica*	*B. rapa*
A4	Baichengbaiyoucai	China	Trib. Brassiceae Hayek	*Brassica*	*B. rapa*
A5	Ling chuan youcai	China	Trib. Brassiceae Hayek	*Brassica*	*B. rapa*
A6	Qu xu	China	Trib. Brassiceae Hayek	*Brassica*	*B. rapa*
A7	Jiningtianjinlv	China	Trib. Brassiceae Hayek	*Brassica*	*B. rapa*
A8	Piaoerbai	China	Trib. Brassiceae Hayek	*Brassica*	*B. rapa*
A9	Niuyezhongshucaixin	China	Trib. Brassiceae Hayek	*Brassica*	*B. parachinensis* L.H.Bailey
A10	Wutacai	China	Trib. Brassiceae Hayek	*Brassica*	*B. narinosa*
A11	Taicai	China	Trib. Brassiceae Hayek	*Brassica*	*B.rapa*
A12	Lelingwuqing	China	Trib. Brassiceae Hayek	*Brassica*	*B.rapa*
A13	Majrova “Petrowski”	Sweden	Trib. Brassiceae Hayek	*Brassica*	*B. rapa*
A14	Majrova “Purple Top Milan”	Sweden	Trib. Brassiceae Hayek	*Brassica*	*B. rapa*
A15	Tradkal“Jersey Walking Stick”	Sweden	Trib. Brassiceae Hayek	*Brassica*	*B. oleracea*
A16	Kalrot Lanttu “Wilhelmsburger”	Sweden	Trib. Brassiceae Hayek	*Brassica*	*B. napus*
A17	Majrova Nauris “Goldhall”	Sweden	Trib. Brassiceae Hayek	*Brassica*	*B. napus*
A18	Midasi	Canada	Trib. Brassiceae Hayek	*Brassica*	*B. napus*
A19	H47	Russia Union	Trib. Brassiceae Hayek	*Brassica*	*B. napus*
A20	Qikuzhen	Japan	Trib. Brassiceae Hayek	*Brassica*	*B. napus*.
A21	Zhongshuan-4	China	Trib. Brassiceae Hayek	*Brassica*	*B. napus*.
A22	Zhongshuan-4NSA	China	Trib. Brassiceae Hayek	*Brassica*	*B. napus*
A23	Baoziganlan	China	Trib. Brassiceae Hayek	*Brassica*	*B. oleracea*
A24	Ziganlan	China	Trib. Brassiceae Hayek	*Brassica*	*B. oleracea*
A25	Shimianlianhuabai	China	Trib. Brassiceae Hayek	*Brassica*	*B. oleracea*
A26	Baipilan	China	Trib. Brassiceae Hayek	*Brassica*	*B. oleracea*
A27	Tuanyexiaohuacai	China	Trib. Brassiceae Hayek	*Brassica*	*B. oleracea*
A28	Lilvqinghuacai	China	Trib. Brassiceae Hayek	*Brassica*	*B. oleracea*
A29	Zhonghuajielan	China	Trib. Brassiceae Hayek	*Brassica*	*B. oleracea*
A30	Yeshengganlan	China	Trib. Brassiceae Hayek	*Brassica*	*B. oleracea*
A31	Yuyiganlan	China	Trib. Brassiceae Hayek	*Brassica*	*B. oleracea*
A32	Nanfangren	Russia Union	Trib. Brassiceae Hayek	*Brassica*	*B. juncea*
A33	Bangcai02	China	Trib. Brassiceae Hayek	*Brassica*	*B. juncea*
A34	Banyedatoucai	China	Trib. Brassiceae Hayek	*Brassica*	*B. juncea*
A35	Donghaigaojiaofengweicai	China	Trib. Brassiceae Hayek	*Brassica*	*B. juncea*
A36	Pusa Bold	India	Trib. Brassiceae Hayek	*Brassica*	*B. juncea*
A37	CMS (Mri)	India	Trib. Brassiceae Hayek	*Brassica*	*B. juncea*
A38	Oiebra	Sweden	Trib. Brassiceae Hayek	*Brassica*	*B. nigra*
A39	Heijie	Germany	Trib. Brassiceae Hayek	Sinapis L.	*B. nigra*
A40	Xinjiangyeshengyoucai A	China	Trib. Brassiceae Hayek	Sinapis L.	*S. arvensis*
A41	Xinjiangyeshengyoucai B	China	Trib. Brassiceae Hayek	Sinapis L.	*S. arvensis*
A42	Baijie	China	Trib. Brassiceae Hayek	Sinapis L.	*S. alba*
A43	Maojiaocaizi	China	Trib. Brassiceae Hayek	*Brassica*	*S. alba*
A44	Ethiopia jie	Ethiopia	Trib. Brassiceae Hayek	*Brassica*	*B. carinata*
A45	Huangziaijie	Ethiopia	Trib. Brassiceae Hayek	*Brassica*	*B. carinata*
A46	77-1304	USA	Trib. Brassiceae Hayek	*Brassica*	*B. carinata*
A47	77-1305	USA	Trib. Brassiceae Hayek	*Brassica*	*B. carinata*
A48	88-212463	USA	Trib. Brassiceae Hayek	*Brassica*	*B. carinata*
A49	88-203221	USA	Trib. Brassiceae Hayek	*Brassica*	*B. carinata*
A50	Yuewangluobozi	China	Trib. Brassiceae Hayek	*Raphanus* L.	*R. sativus*
A51	Lanhuazi	China	Trib. Brassiceae Hayek	*Raphanus* L.	*R. sativus*
A52	Luobozhi (Ogu CMS)	Japan	Trib. Brassiceae Hayek	*Raphanus* L.	*R. sativus*
A53	Eryuelan	China	Trib. Brassiceae Hayek	*Orychophragmus* Bunge	*C. Violaceua*
A54	Celezhahong	China	Trib. Brassiceae Hayek	*Eruca* Mill	*E. sativa*
A55	Yunjie	China	Trib. Brassiceae Hayek	*Eruca* Mill	*E. sativa*
A56	Hancai	China	Trib. Arabideae DC	*Rorippa*	*R. india*
A57	INIA 0863-66	Spain	Trib. Brassiceae Hayek	*Moricandia* DC	*M. arvensis*
A58	Banlangen	China	Trib. Lepidieae DC	*Isatis*	*I. indigotica*
A59	Ziluolan	China	Trib. Matthioleae O.E.Schulz	*Matthiola*	*M. incana*
A60	Tri	Sweden	Trib. Arabideae DC	*Arabidopsis*	*A. thaliana*
A61	KAS	Sweden	Trib. Arabideae DC	*Arabidopsis*	*A. thaliana*
A62	Bsch	Sweden	Trib. Arabideae DC	*Arabidopsis*	*A. thaliana*
A63	mr	Sweden	Trib. Arabideae DC	*Arabidopsis*	*A. thaliana*
A64	Col	Sweden	Trib. Arabideae DC	*Arabidopsis*	*A. thaliana*
A65	Zihuabangguojie	China	Trib. Hesperideae Prantl	*Sterigmostemum* M.Bieb	*S. matthioloides*
A66	Yiguolan	China	Trib. Matthioleae O.E. Schulz	*Diptychocarpus Trautv*	*D. strictus*
A67	Choujie	China	Trib. Lepidieae DC	*Coronopus* J.G.Zinn	*C. didymus*
A68	Dasuanjie	China	Trib. Sisymbrieae DC.	*Sisymbrium* L.	*S. altissimum*
A69	Duoxingdasuanjie	China	Trib. Sisymbrieae DC.	*Sisymbrium* L.	*S. polymorphum*
A70	Xianghuajie	China	Trib. Hesperideae Prantl	*Hesperis* L.	*H. trichosepala*
A71	Ququhua	China	Trib. Lepidieae DC	*Iberis* L.	*I. amara*
A72	Guizhuxiang	China	Trib. Hesperideae Prantl	*Cheiranthus* L.	*Ch. cheiri*
A73	Hancai	China	Trib.Arabideae DC	*Rorippa* Scop.	*R. india*
A74	Keshigaoyuanjie	China	Trib.Arabideae DC	*Christolea* Camb.	*Ch. kashgarica*
A75	Doubancai	China	Trib. Arabideae DC	*Nasturium* R.Br	*N. officinale*
A76	Lizijie	China	Trib. Matthioleae O. E. Schulz	*Chorispora* DC	*Ch. tenella*
A77	Xianyiruijie	China	Trib. Arabideae DC	*Dimorphostemon* Kitag	*D. glandulosus*
A78	Ganxinnianzhujie	China	Trib. Sisymbrieae DC.	*Torularia (Coss)*O.E.Schulz	*T. korolkowii*
A79	Qiganjie	China	Trib. Arabideae DC	*Turritis* L.	*T. glabra*
A80	Sejie	China	Trib. Hesperideae Prantl	*Malcolmia* R.Br	*M. africana*
A81	Suimijie	China	Trib. Arabideae DC	*Cardamine* L.	*C. impatiens*
A82	Songlan	China	Trib. Lepidieae DC	*Iberis* L.	*S. altissimum*
A83	Silengjie	China	Trib. Hesperideae Prantl	*Goldbachia* DC	*G. laevigata*
A84	Xiaoyesuimijie	China	Trib. Arabideae DC	*Cardamine* L.	*C. microzyga*
A85	Tuanshanjie	China	Trib. Alysseae Gren.et Godr	*Berteroa* DC	*B. incana*
A86	Tiaoyetingjie	China	Trib. Alysseae Gren.et Godr	*Alyssum* L.	*A. linifolium*
A87	Xianguojie	China	Trib. Brassiceae Hayek	*Conringia* Adans	*C. planisiliqua*
A88	Xiangxueqiu	China	Trib. Alysseae Gren.et Godr	*Lobularia* Desv.	*L. maritima*
A89	Yanjie	China	Trib. Sisymbrieae DC.	*Thellungiella*	*T. salsuginea*
A90	Xiaoguoyamajie	China	Trib. Sisymbrieae DC.	*Camelina* Crantz	*C. microcarpa*
A91	Ziluolan	China	Trib. Matthioleae O.E.Schulz	*Matthiola* R.Br	*M. incana*

**Table 2:** 90 accessions of cultivars of *B. napus*, and each of 3 accessions of *B. rapa* and *B. oleracea* used for ORG-EcoTILLING.

Sample code	Materials name	Ploidy level	Genome	Origin
B1	Midas	4×	AACC (n = 19)	Canada
B2	Oro	4×	AACC (n = 19)	Canada
B3	Major	4×	AACC (n = 19)	France
B4	Primor	4×	AACC (n = 19)	France
B5	Yaojin Rape	4×	AACC (n = 19)	Italy
B6	Marnoo	4×	AACC (n = 19)	Australia
B7	Ujfertadi	4×	AACC (n = 19)	Hungary
B8	SavariA	4×	AACC (n = 19)	Hungary
B9	Expander	4×	AACC (n = 19)	Germany
B10	Ledos	4×	AACC (n = 19)	Germany
B11	H47	4×	AACC (n = 19)	Russia
B12	H51	4×	AACC (n = 19)	Russia
B13	Janpol	4×	AACC (n = 19)	Poland
B14	Start	4×	AACC (n = 19)	Poland
B15	Mikado	4×	AACC (n = 19)	England
B16	P20	4×	AACC (n = 19)	England
B17	Lingot	4×	AACC (n = 19)	England
B18	Wipot	4×	AACC (n = 19)	Norway
B19	Regent	4×	AACC (n = 19)	Canada
B20	Tower	4×	AACC (n = 19)	Canada
B21	Shiralee	4×	AACC (n = 19)	Australia
B22	Viking	4×	AACC (n = 19)	Denmark
B23	Cobra	4×	AACC (n = 19)	Germany
B24	Parter	4×	AACC (n = 19)	Germany
B25	Falcon	4×	AACC (n = 19)	Germany
B26	Nevin	4×	AACC (n = 19)	France
B27	Samouran	4×	AACC (n = 19)	France
B28	Roman-1	4×	AACC (n = 19)	Netherlands
B29	Tornado	4×	AACC (n = 19)	Sweden
B30	Legend	4×	AACC (n = 19)	Sweden
B31	Grant	4×	AACC (n = 19)	Sweden
B32	Celebra	4×	AACC (n = 19)	Canada
B33	Triton	4×	AACC (n = 19)	Canada
B34	Profit	4×	AACC (n = 19)	Canada
B35	Startigh	4×	AACC (n = 19)	Sweden
B36	Bounty	4×	AACC (n = 19)	Sweden
B37	Garrison	4×	AACC (n = 19)	Sweden
B38	Gcsunder	4×	AACC (n = 19)	Germany
B39	Disamant	4×	AACC (n = 19)	Germany
B40	Mar	4×	AACC (n = 19)	Poland
B41	Star	4×	AACC (n = 19)	Denmark
B42	Shengli Qinggen	4×	AACC (n = 19)	Shanghai, China
B43	Jiuer rape	4×	AACC (n = 19)	Zhejiang, China
B44	Hanfeng-1	4×	AACC (n = 19)	Shanxi, China
B45	Huayou-13	4×	AACC (n = 19)	Wuhan, China
B46	Aijia zao	4×	AACC (n = 19)	Sichuan, China
B47	Southeast-302	4×	AACC (n = 19)	Sichuan, China
B48	Yunyou-49	4×	AACC (n = 19)	Yunnan, China
B49	Qingyou-6	4×	AACC (n = 19)	Qinghai, China
B50	Nonglin-18	4×	AACC (n = 19)	Japan
B51	F01*J6 1-1	4×	AACC (n = 19)	Hubei, China
B52	Ganyou-5	4×	AACC (n = 19)	Wuhan, China
B53	Zhongyou-821	4×	AACC (n = 19)	Wuhan, China
B54	Xiangyou-5	4×	AACC (n = 19)	Hunan, China
B55	Dong-Hae23	4×	AACC (n = 19)	Japan
B56	Ganpol	4×	AACC (n = 19)	Zhejiang, China
B57	Norin16	4×	AACC (n = 19)	Japan
B58	Zheyouyou-2	4×	AACC (n = 19)	Zhejiang, China
B59	Yuyou-2	4×	AACC (n = 19)	Henan, China
B60	Zhongyoudijie-1	4×	AACC (n = 19)	Wuhan, China
B61	Qingyou-12	4×	AACC (n = 19)	Qinghai, China
B62	Qikuzhen	4×	AACC (n = 19)	Japan
B63	Zhongshuang-4	4×	AACC (n = 19)	Wuhan, China
B64	ISN-705	4×	AACC (n = 19)	India
B65	H0302	4×	AACC (n = 19)	Hubei, China
B66	2000-5	4×	AACC (n = 19)	Hubei, China
B67	H9944	4×	AACC (n = 19)	Hubei, China
B68	05 Za-V2	4×	AACC (n = 19)	Chongqing, China
B69	01 Za-654	4×	AACC (n = 19)	Sichuan, China
B70	HY8	4×	AACC (n = 19)	Jiangsu, China
B71	Youyan-10	4×	AACC (n = 19)	Guizhou, China
B72	Qianyou-20	4×	AACC (n = 19)	Guizhou, China
B73	6766	4×	AACC (n = 19)	Hubei, China
B74	H0202	4×	AACC (n = 19)	Hubei, China
B75	Zheyou-5002	4×	AACC (n = 19)	Zhejiang, China
B76	Zhongyouza-2	4×	AACC (n = 19)	Hubei, China
B77	Za-839	4×	AACC (n = 19)	Hunan, China
B78	Hongyou-3	4×	AACC (n = 19)	Jiangsu, China
B79	Zashuang-5	4×	AACC (n = 19)	Henan, China
B80	7633	4×	AACC (n = 19)	Shanxi, China
B81	Qinyou-7	4×	AACC (n = 19)	Shanxi, China
B82	Rape-23	4×	AACC (n = 19)	Shanghai, China
B83	Ganyou-4	4×	AACC (n = 19)	Hubei, China
B84	Huayou-3	4×	AACC (n = 19)	Hubei, China
B85	Huayou-8	4×	AACC (n = 19)	Hubei, China
B86	ChuannongChangjiao	4×	AACC (n = 19)	Sichuan, China
B87	Chuanyou-7	4×	AACC (n = 19)	Sichuan, China
B88	Luzhou-5	4×	AACC (n = 19)	Sichuan, China
B89	Nanyang-41	4×	AACC (n = 19)	Henan, China
B90	F11*J2 1-1	4×	AACC (n = 19)	Hubei, China
B91	Fenyang rape	2×	AA(n = 10)	Shanxi, China
B92	Xishui rape	2×	AA(n = 10)	Hubei, China
B93	Wenjiang Qixingjian	2×	AA(n = 10)	Sichuan, China
B94	2006 Holland-2	2×	CC (n = 9)	Netherlands
B95	Chinese kale yellow/brown seeds	2×	CC (n = 9)	Guangdong, China
B96	C2-7	2×	CC (n = 9)	Spain

### ORG-EcoTILLING procedure

**Figure 1:**
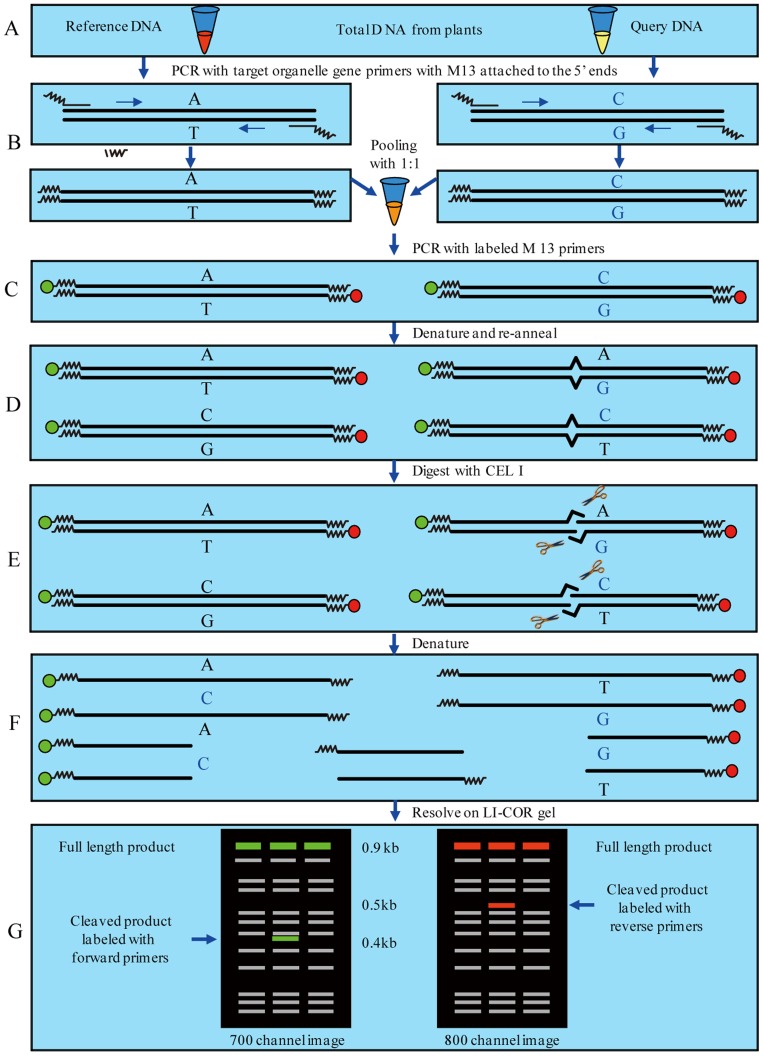
Overview of the ORG-EcoTILLING procedure for detecting DNA polymerphisms in Chloroplast and Mitochondrial genes, modified from Haughn and Gilchrist (2006). A, Total DNA is extracted from plants. B, primers in which a sequence from the universal primer M13F (CACGACGTTGTAAAACGAC) and M13R (GGA TA A CAAT TTCACACAGG) are added to the 5′ end of a target organelle gene primers as adaptors, and query DNA and the reference DNA are amplified, respectively. Then the PCR products of query DNA and reference DNA with the ratio of one to one are pooled. C, the mixture is amplified using the universal primers tagged fluorescently. The forward and reverse primers are labeled with different fluorophors (IRD700 and IRD800) that each label a different end of the PCR fragments. D, the amplified products are denatured by heating and then allowed to cool slowly so that they randomly re-anneal. Heteroduplex molecules form when single DNA strands of mutant and wild-type PCR products anneal together. E, these heteroduplexes become targets for a single-strand-specific nuclease as found in celery juice extract. The nuclease cleaves these heteroduplex fragments at one of the two strands, 3′ to the site of the mismatch in the DNA. F, the PCR products that retain one of the labeled primers can be detected on polyacrylamide denaturing LI-COR gels. G, because the nuclease cleaves either of the two strands randomly, cleavage products can be detected in both the IRD700 and IRD800 channels of the gel image. The position of the mutation within the PCR amplicon can be calculated from the size of the fragments carrying the IRD700-labeled forward primer and the IRD800-labeled reverse primer. The additive size of the cleaved fragments should equal the total length of the entire product.

The ORG-EcoTILLING procedure includes six basic steps (Figure 1), modified from Haughn and Gilchrist (2006) [56]. Firstly, total DNA is extracted from above 181 accessions from the natural population and is normalized. Query DNA and reference DNA are by amplified gene-specific primers with M13F and M13R adaptors, respectively. Then the PCR products of query DNA and reference DNA are mixed in a 1∶1 ratio. The mixture is amplified using a forward primer with 700 nm dye label and a reverse primer with an 800 nm dye label attached to the 5′ ends. Thirdly, the PCR products are heated and cooled to form heteroduplexes between query DNA and reference DNA in the pool. Finally, any mismatches (SNPs or small INDELs) are detected by CEL I (a mismatch endonuclease) which cleaves the heteroduplex into two products, which are detected in the 700 and 800 dye channel of a LI-COR DNA analyzer. The additive size of the two cleaved fragments should equal the total length of the entire product. Because the nuclease cleaves either of the two strands arbitrarily, cleavage products can be detected in both the IRD700 and IRD800 channels of the gel image.

### DNA extraction

Total DNA was extracted from leaves from seedlings according to the methods described by Murray and Thompson [57]. DNA from each accession of 50 pooled plants was normalized to a final concentration of 30 ng·µL^−1^. Following extraction, DNA samples were arrayed in a 96-well format.

### Primer design

Using Primer Premier (PREMIER Biosoft International, http://www.premierbiosoft.com/index.html), two types of primers were designed for PCR amplification: gene-specific primers directly labeled at the 5′ end with IRD 700 and IRD 800 (such as the primer for *atp6*) or primers in which a sequence from the universal primer M13F (CACGACGTTGTAAAACGAC) was added to the 5′ end of a gene-specific primer as an adaptor, and M13R (GGATAACAAT TTCACACAGG) was also added to gene-specific primers (such as the primers for the *accD* gene, *matK* gene, and *rbcL* gene). The universal primers M13F and M13R were labeled at the 5′ end with IRD 700 and IRD 800 separately (MWG Biotech, Inc., Ebersberg, Germany). Altogether, 7 pairs of primers were designed against for 3 genes (*accD*, *matK* and *rbcL*) based on DNA sense strand, except a pair of primer against for *atp6* according to DNA antisense strand. All of the primers are listed in Table 3 and the positions of these primers are shown in Figure S1.

**Table 3:** Primers used in the PCR amplification#.

Taxon	Genes	GenBank No.	Gene length	Primer name	Primer type	Primer sequence	PCR products length
*B. napus B. rapa B.oleracea*	*accD*	GQ861354	1470 bp	accD-1	M13-A1 Forward	M13F-tgactattcatctattgttatt	974 bp
					M13-A1 Reverse	M13R-gctttttataaggttcct	
				accD-2	M13-A2 Forward	M13F-tgaccagttcagttcagagagaatt	789 bp
					M13-A2 Reverse	M13R-tttttcagggttttcgtttaat	
	*matK*	GQ861354	1575 bp	matK-1	M13-M1 Forward	M13F-tgaggtatttagtgctttcg	959 bp
					M13-M1 Reverse	M13R-cttgagcaaccctaagagcg	
				matK-2	M13-M2 Forward	M13F-gcaagcagtcttctcatttacg	948 bp
					M13-M2 Reverse	M13R-ttttgttatctccgcattcc	
	*rbcL*	GQ861354	1440 bp	rbcL-1	M13-R1 Forward	M13F-caacatatattactgtcaagag	974 bp
					M13-R1 Reverse	M13R-ttcacattctcatcatctttgg	
				rbcL-2	M13-R2 Forward	M13F-cagtttatgaatgtctacgtgg	1081 bp
					M13-R2 Reverse	M13R-cttctttttttttcagattttg	
Cruciferae	*accD*	AP000423	1476 bp	CA	M13-CAForward	M13F-tgaaagttacatttataatt	827 bp
					M13-CAReverse	M13R-cttttttcaatgtttgttca	
	*atp6*	NM_126768.1	786 bp	UPA	IRD700 Forward	700*-ggagatttatagcatcattcaag	733 bp
					IRD800 Reverse	800*-attgtcccattgattcctat	

# Universal primers in this study included M13-IRD700 Forward and M13-IRD800 Reverse, and the corresponding primer sequences were 700*-cacgacgttgtaaaacgac and 800*-ggataacaatttcacacagg, respectively.

### PCR amplification and mutation detection

PCR amplification and mutation detection followed the method of Barkley and Wang [58] with some modifications. For the *accD*, *matK* and *rbcL* genes, we used a two-step approach to amplify DNA because the primer pairs carried the universal primers M13F and M13R. The first PCR amplification was performed in a 15 µL reaction volume using 60 ng DNA template, 1.5 µL 10× Ex-buffer, 0.2 mM dNTPs, 0.75 U Ex-Taq polymerase and 0.25 µL 10 µM each primer [Ex-Taq polymerase and Ex-buffer both purchased from TAKARA Biotechnology (Dalian) CO., LTD.]. Cycling conditions were as follows: 94°C for 5 min, 35 cycles of 94°C for 1 min, primer-specific annealing temperature for 1 min (the annealing temperatures for the *accD*, *matK* and *rbcL* genes were 50°C, 55°C and 57°C, respectively) and 72°C for 1 min, followed by a final extension for 10 min at 72°C. After the first PCR amplification, 1 µL of the PCR products was diluted to a 50-µl volume, and then 2 µL of the diluted PCR products was extracted, mixed with 2 µL of the control PCR sample to serve as a template for the second PCR amplification. The reference samples of *accD*, *matK* and *rbcL* in *Brassica* species were B26, B26 and B85, respectively. However, the reference sample of *accD* in Brassiceae was A64. The second PCR was performed in 25-µL reaction volume using 2.5 µL 10× Ex-buffer, 0.32 mM dNTPs, 1.25 U Ex-Taq polymerase, and 1.0 µL 1 µM M13F and M13R universal primers labeled at the 5′ end with IRD 700 and IRD 800. PCR amplifications were conducted using the same reaction program as for the first PCR, except that 55°C was used for the annealing temperature. However, for the *atp6* gene, PCR amplification was performed in the same way as the first PCR amplification of the two-step approach, except that 0.25 µL 1 µM each primer directly labeled with IRD 700 and IRD 800 was used. Sample A24 was served as the reference DNA in the *atp6* gene of Brassicaceae.

After PCR amplification, denaturation, slow reannealing conditions were applied to the PCR fragments to form heteroduplexes. The products were denatured and annealed in a thermal cycler, as follows: 99°C for 10 min, 70 cycles of 70°C for 20 sec, with a decrement of 0.5°C per cycle, and finally, 8°C for 5 min. A total of 30 µL of the processed PCR fragments was incubated in 2 µL of 10×CEL I buffer (10×CEL I buffer include 5 mL 1 M MgSO4, 100 µL 10% Triton X-100, 5 mL 1 M Hepes (pH 7.4), 5 µL 20 mg/ml bovine serum albumen, 2.5 ml 2 M KCl, 37.5 ml ddH_2_O), 0.2 µL purified CEL I extracted and purified according to the method described by Oleykowski et al (1998) [59] and 10 µL of heteroduplexes at 45°C for 30 min. CEL I cuts with partial efficiency, allowing the detection of multiple mismatches in a DNA duplex [48]. The CEL I digestion reaction was stopped with 5 µL of 0.225 mM EDTA. Sample purification was performed using Sephadex G50 (medium coarse) columns, and 2 µL of blue stop solution (MWG Biotech, Inc., Ebersberg, Germany) was added to each sample. Then, all of the samples were concentrated to a final volume of 2–3 µL for 60 min at 85°C. A total of 0.8 µL was loaded onto 6.5% polyacrylamide gels on a LI-COR 4300 DNA analyzer. To confirm the polymorphisms identified by ORG-EcoTILLING, each gel image of ORG-EcoTILLING was generated from two replicate rans. Because the fragment patterns for the two replicates were always concordant, we selected the better quality gel image for analysis. Furthermore, the accessions exhibiting polymorphism on the gels were randomly selected for a second round of amplification with the corresponding primer pairs using proofreading Ex-Taq polymerase. The PCR products were sequenced with an ABI 3730 and chromatograms checked to identify PCR or sequencing errors.

### Mutation frequency and detection rate

A total of 141,120 bp, 151,200 bp and 138,240 bp were screened for the *accD*, *matK* and *rbcL* genes separately, which was calculated by the multiplication of the number of samples and the total length of the gene sequence. The mutation frequency was determined by dividing the total base pairs screened by the total mutants detected. The detection rate was calculated by the following formula: Detection rate = A1/A2 (A1 was the number of mutations detected by ORG- EcoTILLING, and A2 was the number of mutations checked by sequencing).

### Phylogenetic tree construction

To construct the phylogenetic tree for chloroplast genes, *accD*, *matK* and *rbcL* sequences of 90 accessions of *B. napus* and each of 3 accessions of *B. rapa* and *B. oleracea* were generated (GenBank accession numbers JF807893 to JF807913 inclusive), CLUSTAL W, version 1.81 [60] was used for sequence alignment and Phylip 3.68 with a neighbour-joining (N-J) algorithm (http://evolution.genetics.washington.edu/phylip.html) [61] for phylogenetic and molecular evolutionary analyses. The evolutionary distance (D) was computed using Kimura's two-parameter method [62], and the trees tested with 1000 bootstrap replicates. The corresponding bootstrap values (>750) for each data partition were displayed in each node on the tree.

## Results

### Applicability of ORG-EcoTILLING in the discovery of chloroplast DNA polymorphism for inter- and intra-specific analysis of *Brassica*

**Figure 2:**
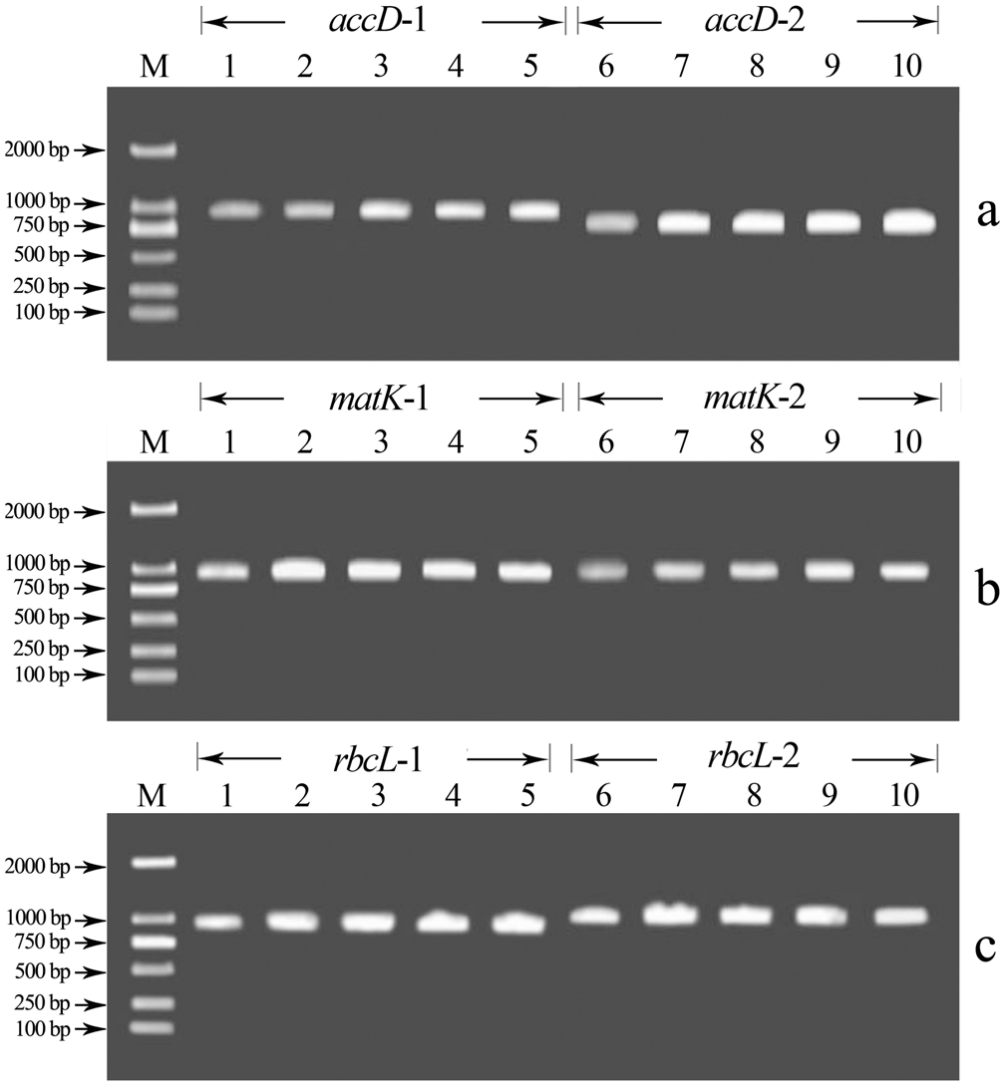
Detection of amplified fragments of selectcd chloroplast genes by agarose gel electrophoresis. a. *accD* gene, the size of PCR products in *accD*-1 and *accD*-2 amplified by two pairs of primers were 974 and 789 bp, respectively. b. *matK* gene, the size of PCR products in *matK*-1 and *matK*-2 amplified by two pairs of primers were 959 and 948 bp, respectively. c. *rbcL* gene, the size of PCR products in *rbcL*-1 and *rbcL*-2 amplified by two pairs of primers were 974 and 1081 bp, respectively. The sizes of the DNA fragments produced for the three genes corresponded with what we expected, and the abundance of DNA was fit for ORG-EcoTILLING analysis. The samples from 1 to 5 were B12, B20, B26, B85 and B94 in *accD*-1 gene fragment, and the same in other gene fragments. The molecular size of DNA is shown at the left with arrowheads. M represented the DNA ladder DL 2000 (Promega. Inc).

A total of 90 *B. napus* samples and three each of *B. rapa* and *B. oleracea* were analyzed. The *accD*, *matK* and *rbcL* genes were amplified using two pairs of primers each. Amplified DNA fragments were of expected length for the three genes, as detected by agarose gel electrophoresis (Figure 2), and the DNA yield was suitable for ORG-EcoTILLING analysis. The analysis reliably located the position of mutated base pairs in the *accD*, *matK* and *rbcL* genes.

**Figure 3:**
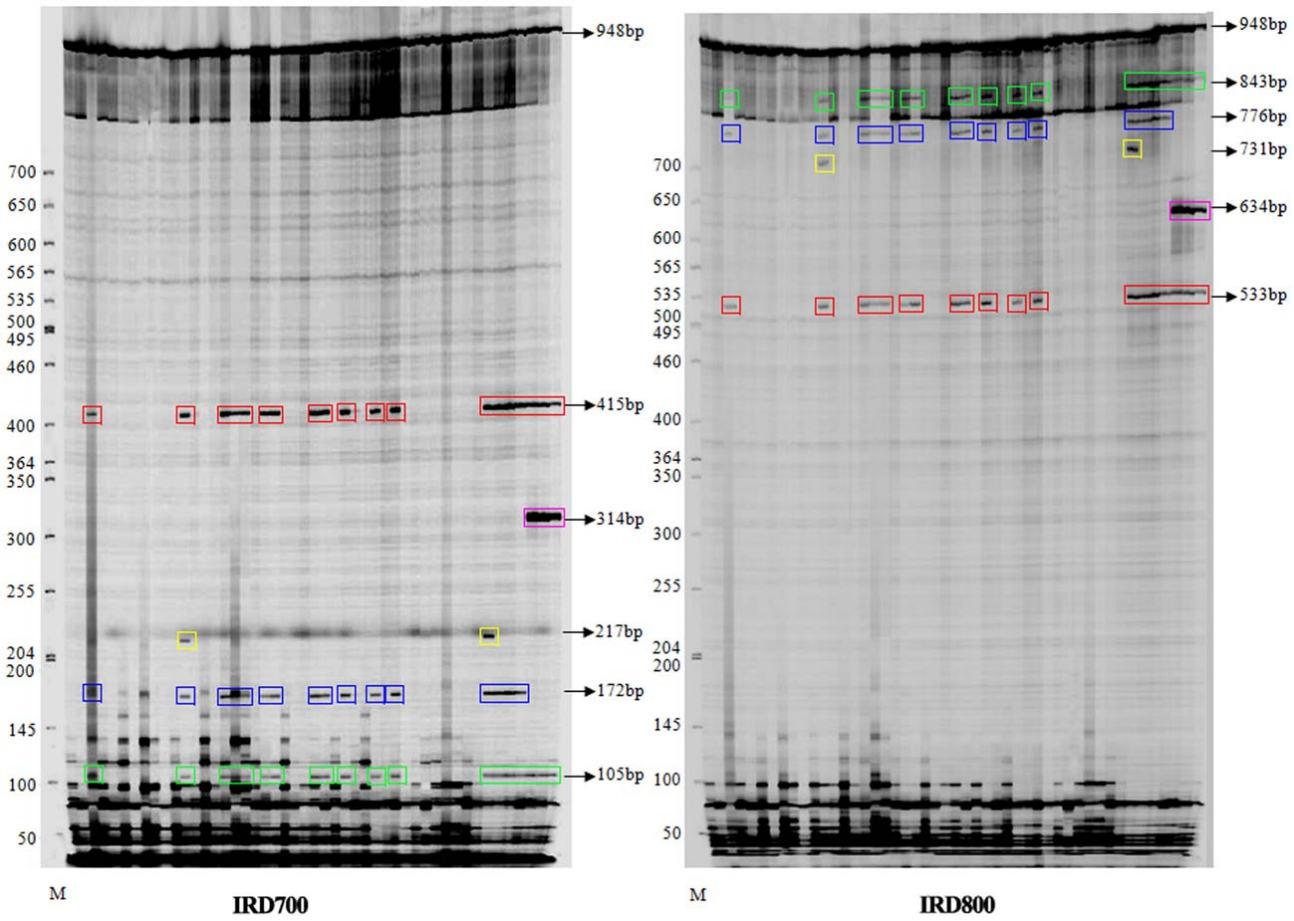
Overview of mutation detections in *matK* gene of *matK*-2 fragment by ORG- EcoTILLING. Samples were resolved by denaturing polyacrylamide gel electrophoresis and visualized with the LI-COR DNA analyzer. The sample in each lane from left to right was B49–B96 samples. The IRD 700 and IRD 800 channels were shown respectively. CEL I-cleaved heteroduplexes appeared as intense bands (circled in different color rectangles of IRD 700 channel and corresponding same color ones of IRD 800 channel). The size of the specific cleavage products in the IRD700 labeled plus the size of the IRD800 labeled (indicated by arrowheads in the same color geometries, respectively) was equal to that of uncleaved fragment (948 bp). The fragment sizes also indicated the position of the mutation present in the locus *matK* gene of the representative samples. The reference sample was B26.

As an example, the IRD 700 and IRD 800 channels for the *matK* gene fragment (Primer: M13-M2 Forward and M13-M2 reverse, Table 3) are shown in Figure 3. This demonstrates that mismatches were detected by CEL I and cleaved into two separate products, which were distinctly detected in both the IRD700 and IRD800 channels of the gel image. The combined lengths of the IRD700-labeled and IRD800-labeled cleaved fragments corresponded to the length of the PCR fragments (948 bp) in the *matK* gene. Similarly, clear gel images were obtained for the *accD* and *rbcL* genes, and the IRD700-labeled and the combined length of the IRD800-labeled cleaved fragments corresponded to the PCR fragments of the two genes (Table S1).

ORG-EcoTILLING detected nine, six and a single distinct mutated base pair positions in the *accD*, *matK* and *rbcL* genes, respectively, corresponding to 25, 25 and 19 mutated out of the 96 samples (Table S1). Two primer pairs were designed to amplify each of the three genes; their target regions were denominated *accD*-1, *accD*-2, *matK*-1, *matK*-2, *rbcL*-1 and *rbcL*-2, respectively (Table 3). For the *accD* gene, most samples had one or two mutation points within *accD*-1, whereas there were five mutation points in *accD*-2, such as in samples B94, B95 and B96, *B. oleracea*, the ancestral parents of *B. napus*. In contrast, three or four mutation points were present in *matK*-2, but only one mutation point was found in *matK*-1. For the *rbcL* gene, a single mutation was observed in *rbcL*-1, and none in *rbcL*-2.

Interestingly, the three varieties of *B. rapa* possessed the same mutation positions as *B. napus* in the *accD*, *matK* and *rbcL* genes, respectively, whereas different mutation positions in the *accD* and *matK* genes were discovered in the three varieties of *B. oleracea*. For example, samples B94, B95 and B96 all had specific mutation positions in the *accD*-1 gene fragment, such as positions 409 and 871 in the IRD700 channel and five mutation positions in the *accD*-2 gene fragment, which were not observed in the *B. napus* and *B. rapa* varieties. Similarly, although the mutation positions in the *matK*-2 gene fragment were approximately the same in all mutation samples, only the three samples of *B. oleracea* presented one mutation position each in the *matK*-1 gene fragment. For the *rbcL* gene, the three samples of *B. oleracea* showed no mutation. Moreover, the samples mutated in the *accD* gene corresponded with those mutated in the *matK* gene, except sample B68. Similarly, the samples mutated in the *rbcL* gene were, without exception, distributed around the samples mutated of the *accD* and *matK* genes. Thus, our results effectively indicated that ORG-EcoTILLING could be effectively applied in chloroplast genes and that ORG-EcoTILLING could detect chloroplast DNA polymorphisms among the three species of *Brassica*, and very importantly among varieties of *B. napus*.

### Application of ORG-EcoLLING for detecting SNPs within the *accD* gene across the Brassicaceae family

The key chloroplast gene *accD* was used to study the application of ORG-EcoTILLING in members of the Brassicaceae family (91 samples of different taxa). We designed the primers for the *accD* gene from a conservative region according to BLAST searches of different Brassicaceae taxa. The size of the *accD* fragment amplified by PCR was 827 bp in all samples.

**Figure 4:**
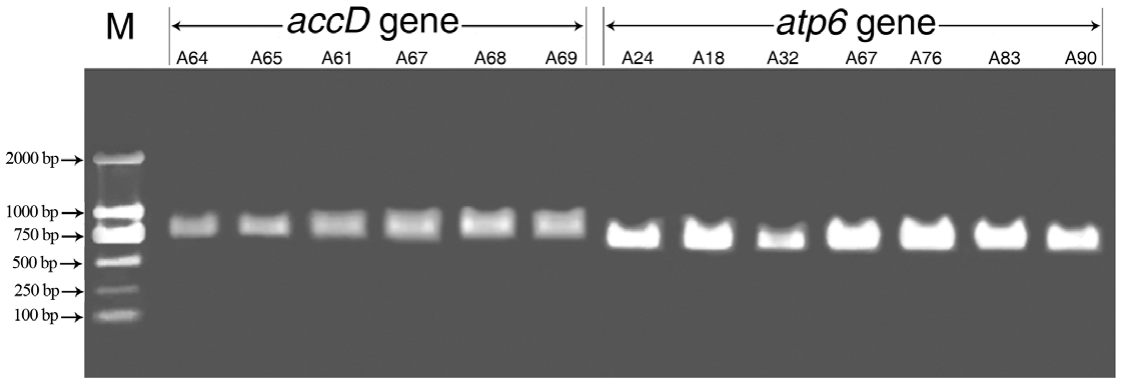
Examples of DNA amplified regions detection of plastidic genes separated by agarose gel electrophoresis. The picture shows the results from PCR analyses of plastidic gene *accD* (primer pair: M13-CAF and M13-CAR), and *atp6* (primer pair: M13-CP F and M13-CP R), respectively. The samples of *accD* gene and *atp6* came from 91 various species in Cruciferae. The sizes of the DNA fragments in the two genes detected by agarose gel electrophoresis were consistent with the result of primers design, and the abundance of DNA was fit for ORG-EcoTILLING analysis. The molecular size of DNA is shown at the left with arrowheads. M represented the DNA ladder DL 2000 (Promega. Inc).

**Figure 5:**
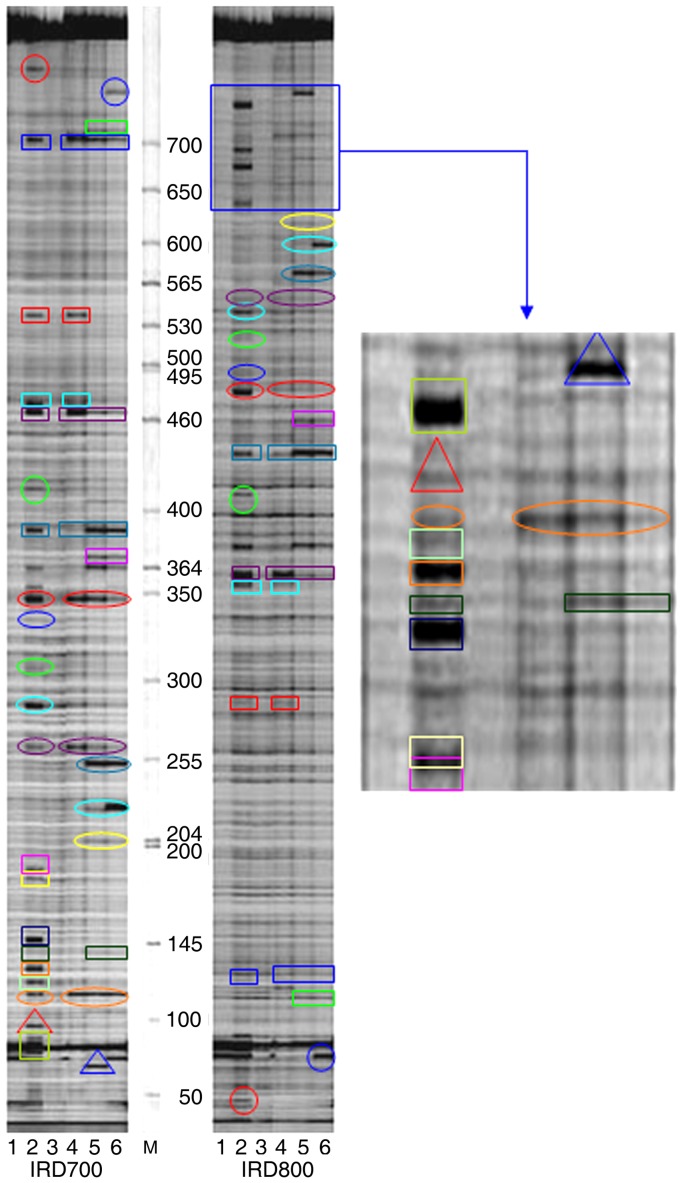
Overview of mutation detections in Brassicaceae samples by ORG- EcoTILLING Electrophoresis on a 6.5% KBplus Gel of CEL I-digested products of APCR fragment of *accD* gene, 827 bp in ALI-COR 4300 DNA Analyzer were displayed here. The sample in each lane from one to six was A64, A65, A61, A67, A68 and A69, respectively. CEL I-cleaved heteroduplexes appeared as intense bands (circled in different color geometric figures of IRD 700 channel and corresponding same color ones of IRD 800 channel). The fragment sizes indicated the position of the mutation present in the locus *accD* gene of the collected samples. The reference sample in this gene was A64.

Sufficient DNA was generated for subsequent analysis (Figure 4). Overall, 44 mutation points in the *accD* gene were detected in 44 plant samples (Table S2). Analysis of a subset of CEL 1 digests in the representative samples (Figure 5) revealed sample A61 and the reference sample A64 both lacked cleaved positions, whilst samples A65, A67, A68 and A69 were characterized by 29, 9, 15 and 16 mutation sites, respectively. Furthermore, it was clear that the sizes of the 2 fragments produced for all mutation points indicated the accurate position of the mismatch, and thus the site of the mutation or nucleotide change. Detailed ORG-EcoTILLING analysis for these four mutation samples is shown in Table 4. Distinct SNPs were observed in the *accD* gene of the Brassicaceae family. For instance, these four mutation samples all shared with mutation site 6, 20, 25, 29, 32 and 37. However, sample A65 had 20 specific polymorphic sites which were not shared with other samples (Table 4). The results demonstrated that ORG-EcoTILLING could not only be applied for detecting DNA polymorphisms in chloroplast genes in different tribes and genera, but also among and between species.

**Table 4:** Analysis of ORG-EcoTILLING information in 700 and 800 channel from Figure 5.

Mutation site No.	Geometric figure	IDY 700	IDY 800	Molecular Size (bp)	Sample code.
1	blue triangle	73	754	827	A68
2	lime square	86	741	827	A65
3	lime square	87	740	827	A65
4	lime square	88	739	827	A65
5	red triangle	100	727	827	A65
6	orange ellipse	119	708	827	A65, A67,A68, A69
7	light green rectangle	127	700	827	A65
8	orange rectangle	134	693	827	A65
9	orange rectangle	135	692	827	A65
10	green rectangle	144	683	827	A65, A68, A69
11	blue rectangle	151	676	827	A65
12	blue rectangle	152	675	827	A65
13	yellow rectangle	187	640	827	A65
14	purple rectangle	191	636	827	A65
15	yellow ellipse	207	620	827	A68, A69
16	turquoise circular	225	602	827	A68, A69
17	turquoise circular	227	600	827	A69
18	cyan ellipse	250	577	827	A68, A69
19	cyan ellipse	251	576	827	A68, A69
20	dark purple ellipse	260	567	827	A65, A67, A68, A69
21	turquoise ellipse	283	544	827	A65
22	emerald green ellipse	305	522	827	A65
23	blue ellipse	339	488	827	A65
24	red ellipse	345	482	827	A65
25	red ellipse	346	481	827	A65, A67, A68, A69
26	purple rectangle	372	455	827	A68, A69
27	cyan rectangle	388	439	827	A65
28	cyan rectangle	388	439	827	A68, A69
29	cyan rectangle	389	438	827	A65, A67, A68, A69
30	green circular	416	411	827	A65
31	dark purple rectangle	468	359	827	A65, A67
32	dark purple rectangle	469	358	827	A65, A67, A68, A69
33	dark purple rectangle	470	357	827	A65
34	turquoise rectangle	474	353	827	A65
35	turquoise rectangle	474	353	827	A67
36	red rectangle	543	284	827	A65, A67
37	blue rectangle	704	123	827	A65, A67, A68, A69
38	emerald green rectangle	713	114	827	A68, A69
39	blue circular	755	72	827	A69
40	red circular	781	46	827	A65

The sizes of the two fragments of IRD 700 and 800 indicated the accurate position of the mismatch. Descriptions of different color geometric figures were corresponding with that of Figure 5.

### Application of ORG-EcoTILLING for the detecting of SNPs within the *atp6* gene across the Brassicaceae family

**Figure 6:**
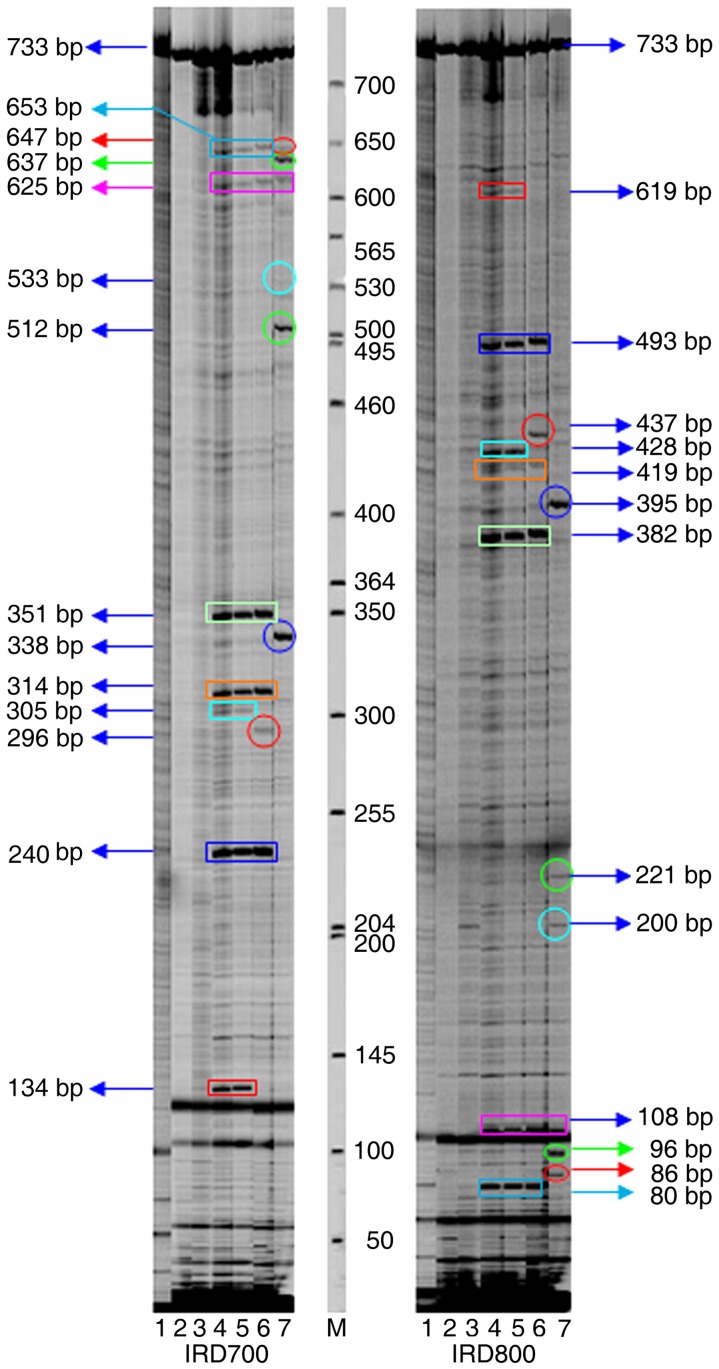
Images of ORG-EcoTILLING gels of CEL I-digested products of a PCR fragment of *atp6* gene obtained from each of the two fluorescent channels of the LI-COR 4300 DNA Analyzer. The intense bands (circled in different color geometric figures of IRD 700 channel and corresponding same color ones of IRD 800 channel) came from the product of CEL I-cleaved heteroduplexes. The sample in each lane from left to right was A24, A18, A32, A67, A76, A83 and A90, respectively, and sample A24 acted as the reference sample of *atp*6 gene for ORG-EcoTILLING. Therefore, it was obvious that 13 polymorphism sites were detected in *atp6* gene among these seven samples.

A key mitochondrial gene, *atp6*, was chosen to study the application of ORG-EcoTILLING in 91 different taxa of Brassicaceae. Initially, the primer for the *atp6* gene was designed from a conserved region based on BLAST searches of different Brassicaceae taxa, with an expected PCR product length of 733 bp, which was confirmed in all 91 accessions by agarose gel electrophoresis (Figure 4). Analysis of all products of CEL 1 digests indicated that there were 17 mutation points distributed in 18 plant samples (Table 5). The distribution of the mutation points in the *atp6* gene indicated that this atp6 gene was conserved amongst genera of Trib. Brassiceae, since only three mutation points were observed in sample A87, but no point mutations in other Trib. Brassiceae accession. In contrast, abundant mutations of the atp6 gene were detected in Trib. Lepidieae and Trib. Sisymbrieae, the former shared 11 mutation points among four plant accessions and the latter having eight SNPs in two plant samples. In addition, seven mutation points were detected in both of Trib. Hesperideae and Trib. Matthioleae, six SNPs in Trib. Arabideae and four polymorphic sites in Trib. Alysseae. It should be noted that there are distinctly different in mutation sites within *atp6* gene among different genus or species of the same tribes. ORG- EcoTILLING images of seven samples (Figure 6) revealed that 13 nucleotide mutations were detected in the atp6 locus in samples A67, A76, A83 and A90, while samples A18, A24 and A32 lacking cleaved positions. Therefore, it was clearly shown that ORG- EcoTILLING could be applied to the detection of mutations in a mitochondrial gene.

**Table 5:** Band analysis of *atp6* gene in the family of Brassicaceae identified by ORG- EcoTILLING.

	MW (bp) IRD700																
Mutated sample code	134	140	240	296	305	314	338	351	368	465	512	533	625	637	647	653	663
A56											+				+		
A58													+		+		+
A60		+							+		+		+		+		
A61		+							+		+		+		+		
A67	+		+		+	+		+					+			+	
A68	+															+	
A70																+	
A71	+												+		+		
A72															+		
A73											+	+	+		+		
A75									+		+	+	+			+	
A76	+		+		+	+		+					+			+	
A79											+	+	+				
A82													+		+		+
A83			+	+		+		+					+			+	
A87													+		+		+
A88	+									+			+			+	
A90							+				+	+	+	+	+		

“+” indicated that there was an intense band visualized by denaturing polyacrylamide gel electrophoresis with the LI-COR DNA analyzer.

### Validation of the accuracy of ORG-EcoTILLING for the identification of cytoplasmic DNA Polymorphism

We validated the accuracy of ORG-EcoTILLING by Sanger DNA sequencing. At the species level for the *accD*, *matK* and *rbcL* genes in 96 samples of *B. napus*, *B. rapa* and *B. oleracea*, mutation points were detected in 26, 26, and 19 samples, respectively. However, 70, 70 and 77 samples of *B. napus* presented no mutation points in the corresponding genes, *accD*, *matK* and *rbcL* by ORG-EcoTILLING. According to the Sanger sequencing results, 116, 89 and 19 DNA polymorphisms were detected in *accD*, *matK* and *rbcL* genes, respectively, and the results of ORG-EcoTILLING were entirely consistent with those of DNA sequencing results (Table 6). In addition, we calculated that the *accD* gene in *B. napus*, *B. rapa* and *B. oleracea* had approximately one mutation per 1.22 kb, compared with approximately one mutation per 1.70 kB and 7.28 kB in the *matK* and *rbcL* genes, respectively (Table 6). We then analyzed SNP classes based on the results of DNA sequencing. Two SNP classes of deletion and transition mutations were detected in the three genes, with the deletion type only presented in the *accD* gene (in the form of six nucleotide base pair deletions). However, the majority of the transitions detected were C to A in the *accD* gene; G to T, T to G and T to A in the *matK* gene; and only G to A transitions in the *rbcL* gene. All sequence variants detected by DNA sequencing were also detected by ORG-EcoTILLING. Therefore, the detection rate of ORG- EcoTILLING was 100%.

**Table 6:** SNP mutation types and band analysis of *B. napus* varieties identified by ORG- EcoTILLING and DNA sequencing.

Sample code	*accD* mutation position									*matK* mutation position						*rbcL* mutation position
	216 (G/T)*	335 (T/A)	560–565 (AAAGTG)	644 (A/C)	797 (C/T)	858 (G/A)	1058 (G/A)	1177 (T/G)	1275 (T/C)	201 (A/G)	822 (T/A)	889 (T/G)	934 (G/A)	1031–1032 (TT/AA)	1132 (G/T)	66 (G/A)
B11			+	+							+	+			+	+
B12				+							+	+			+	+
B14	+		+	+							+	+	+		+	+
B25			+	+											+	
B27			+	+							+	+			+	+
B29			+	+							+	+	+		+	
B32				+							+	+			+	+
B45			+	+							+	+			+	
B51			+	+							+	+			+	+
B60	+		+	+							+	+	+		+	+
B64				+							+	+			+	+
B65				+							+	+			+	+
B66				+							+	+			+	+
B68											+	+			+	+
B69				+							+	+			+	
B73				+							+	+			+	+
B74				+							+	+			+	+
B76				+							+	+			+	+
B79				+							+	+			+	
B81				+							+	+			+	+
B90	+		+	+							+	+	+		+	+
B91			+	+							+	+			+	+
B92			+	+							+	+			+	+
B93			+	+							+	+			+	+
B94		+			+	+	+	+	+	+	+			+	+	
B95		+			+	+	+	+	+	+	+			+	+	
B96		+			+	+	+	+	+	+	+			+	+	
	3	3	72	23	3	3	3	3	3	3	26	23	4	6	27	19
Total	116									89						19
sequencing	116									89						19
Detection rate	100%									100%						100%
Total size	141120 bp									151200 bp						138240 bp
Frequency	1/1.22 kb									1/1.70 kb						1/7.28 kb

Note: The sample codes were corresponding with Table 2, and mutation position was located from initiation site (ATG) of a gene. The length of *accD*, *matK* and *rbcL* were 1470 bp, 1575 bp and 1440 bp, respectively. “+” indicated that there was an intense band visualized by denaturing polyacrylamide gel electrophoresis with the LI-COR DNA analyzer.

* The base pairs changes in round bracket were detected by DNA sequencing and G to T change, while “AAAGTG” meant deletion of base pairs. The reference sample of *accD*, *matK* and *rbcL* were B26, B26 and B85, respectively.

At the intertribal, intergeneric and interspecific levels, 40 and 13 mutation positions were detected by ORG-EcoTILLING from the total of 13 samples screened for the *accD* and *atp6* gene fragments, respectively (Figure 5 and 6). Although we identified only one type of mutation (base substitution) in the *accD* and *atp6* genes, there were abundant SNP types in these mutations (Table 7, Figure S2 and S3). There were 37, 15, 27 and 29 polymorphism sites (a total of 108 polymorphic sites)in *accD* gene among sample A65, A67, A68 and A69, respectively, and only 29, 9, 15 and 16 mutation sites (a total of 69 polymorphic sites) were discovered in these corresponding samples by ORG-EcoTILLING. Therefore, the detection rate within this gene was 64%. Two particular discrepancies primarily contributed to the low detection rate of ORG-EcoTILLING. Within the first 80 bp only one out of 13 polymorphic sites was detected. Additionally, between nucleotides 354 and 380 within *accD* gene in A65, A67, A68 and A69, there were 14 polymorphism sites within a range of 27 bases (Table 7, Figure S2), but only two of these were detected compared with the results of DNA sequencing. In contrast, all 13 polymorphic sites representing different types of SNPs within *atp6* gene fragments were detected using ORG-EcoTILLING (Table 7), thus the detection rate within this gene was 100%.

**Table 7:** Comparison of efficiency in different types of SNPs in *accD* and *atp6* gene detected by ORG-EcoTILLING and DNA sequencing.

positions of polymorphisms		SNPs detected by sequencing					SNPS detected by ORG-EcoTILLING					Detection rate (%)
		A64*	A65	A67	A68	A69	A64*	A65	A67	A68	A69	
*accD* gene	56	C	T	T	T	T	NP	−	−	−	−	0
	63	A	A	A	G	G	NP	NP	NP	−	−	0
	73	G	G	G	A	G	NP	NP	NP	+	NP	100
	74	T	C	C	C	C	NP	−	−	−	−	0
	81	T	T	T	G	G	NP	NP	NP	−	−	0
	86	G	A	G	G	G	NP	+	NP	NP	NP	100
	87	A	C	A	A	A	NP	+	NP	NP	NP	100
	88	T	A	T	T	T	NP	+	NP	NP	NP	100
	100	G	T	G	G	G	NP	+	NP	NP	NP	100
	109	G	G	G	A	A	NP	NP	NP	−	−	0
	119	A	G	G	G	G	NP	+	+	+	+	100
	127	A	G	A	A	A	NP	+	NP	NP	NP	100
	134	C	A	C	C	C	NP	+	NP	NP	NP	100
	135	G	T	G	G	G	NP	+	NP	NP	NP	100
	144	C	A	C	A	A	NP	+	NP	+	+	100
	151	A	C	A	A	A	NP	+	NP	NP	NP	100
	152	C	T	C	C	C	NP	+	NP	NP	NP	100
	187	G	T	G	G	G	NP	+	NP	NP	NP	100
	191	A	G	A	A	A	NP	+	NP	NP	NP	100
	207	A	A	A	G	G	NP	NP	NP	+	+	100
	225	C	C	C	G	G	NP	NP	NP	+	+	100
	227	A	A	A	A	G	NP	NP	NP	NP	+	100
	250	G	G	G	C	C	NP	NP	NP	+	+	100
	251	T	T	T	C	C	NP	NP	NP	+	+	100
	260	A	G	G	G	G	NP	+	+	+	+	100
	266	C	T	C	T	T	NP	−	NP	−	−	0
	283	C	G	C	C	C	NP	+	NP	NP	NP	100
	305	T	C	T	T	T	NP	+	NP	NP	NP	100
	339	C	A	C	C	C	NP	+	NP	NP	NP	100
	345	A	C	A	A	A	NP	+	NP	NP	NP	100
	346	A	G	G	G	G	NP	+	+	+	+	100
	354	C	A	C	C	T	NP	−	NP	NP	−	0
	365	C	A	T	G	G	NP	−	−	−	−	0
	368	T	T	T	C	C	NP	NP	NP	−	−	0
	372	G	G	G	A	A	NP	NP	NP	+	+	100
	380	G	T	T	T	T	NP	−	−	−	−	0
	388	C	T	C	A	A	NP	+	NP	+	+	100
	389	T	G	G	G	G	NP	+	+	+	+	100
	395	A	A	A	G	G	NP	NP	NP	−	−	0
	408	G	G	G	A	A	NP	NP	NP	−	−	0
	416	A	C	A	A	A	NP	+	NP	NP	NP	100
	449	C	C	C	T	T	NP	NP	NP	−	−	0
	450	C	A	C	C	C	NP	−	NP	NP	NP	0
	468	C	A	A	C	C	NP	+	+	NP	NP	100
	469	G	A	A	A	A	NP	+	+	+	+	100
	470	G	T	G	G	G	NP	+	NP	NP	NP	100
	474	A	C	T	A	A	NP	+	+	NP	NP	100
	522	A	G	A	A	A	NP	−	NP	NP	NP	0
	543	T	C	C	T	T	NP	+	+	NP	NP	100
	704	A	G	G	G	G	NP	+	+	+	+	100
	707	G	G	C	G	G	NP	NP	−	NP	NP	0
	713	T	T	T	A	A	NP	NP	NP	+	+	100
	746	C	C	T	C	C	NP	NP	−	NP	NP	0
	755	T	T	T	T	C	NP	NP	NP	NP	+	100
	781	G	T	G	G	G	NP	+	NP	NP	NP	100
Total mutation sites			108 69									
Average Detection rate (%)			64									
*atp6* gene		A24*	A67	A76	A83	A90	A24*	A67	A76	A83	A90	
	134	G	T	T	G	G	NP	+	+	NP	NP	100
	240	G	C	C	C	G	NP	+	+	+	NP	100
	296	T	T	T	C	T	NP	NP	NP	+	NP	100
	305	T	C	C	T	T	NP	+	+	NP	NP	100
	314	T	G	G	G	T	NP	+	+	+	NP	100
	338	A	A	A	A	G	NP	NP	NP	NP	+	100
	351	C	G	G	G	C	NP	+	+	+	NP	100
	512	T	T	T	T	C	NP	NP	NP	NP	+	100
	533	T	T	T	T	C	NP	NP	NP	NP	+	100
	625	T	C	C	C	C	NP	+	+	+	+	100
	637	G	G	G	G	A	NP	NP	NP	NP	+	100
	647	A	A	A	A	T	NP	NP	NP	NP	+	100
	653	C	A	A	A	C	NP	+	+	+	NP	100
Average Detection rate (%)			100									

* indicated that the reference sample was A64 and A24 in *accD* gene and *atp6* gene, respectively. “+” indicated that there was an intense band visualized by ORG-EcoTIILLING. “−” indicated that there was no intense band detected by ORG-EcoTIILLING. “NP” indicated no polymorphism.

Very interestingly, we observed that the same polymorphic position detected by ORG-EcoTILLING revealed distinct transitions in different samples for the *accD* gene. For example, based on sequencing results, at position 365, base C mutated to base A, T and G among these samples, and for position 474, base A mutated to base C and T (Table 7, Figure S2). The latter was detected by ORG-EcoTILLING, while the former not.

**Figure 7:**
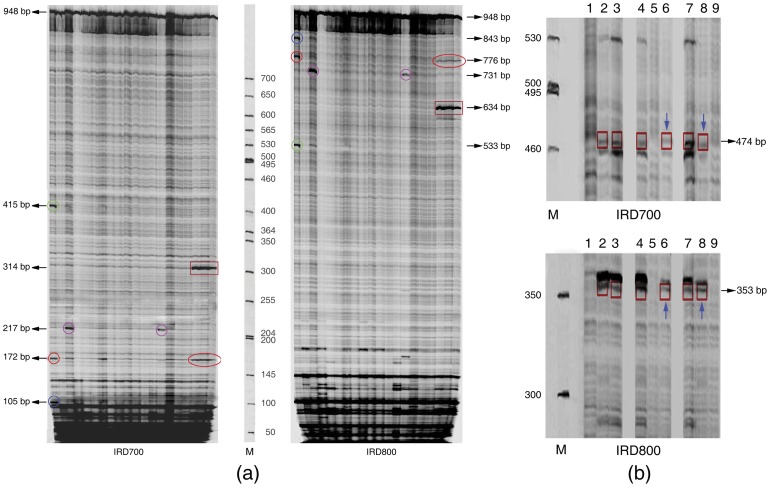
Gel image showing the result of validation in the difference of the same polymorphic position discovered by ORG-EcoTILLING among *Brassica* species or Cruciferae. (a) Based on the results of ORG-EcoTILLING in matK-2 fragment of *Brassica* intraspecies in Figure 3, 19 mutated samples and no mutation sample B26 (as the reference in Figure 3) were selected to mix with B76 (as the reference), respectively. The sample in each lane from left to right in the channel of IRD700 or IRD800 was B26, B51, B60, B64, B65, B66, B68, B69, B73, B74, B76, B79, B81, B90, B91, B92, B93, B94, B95 and B96. CEL I-cleaved heteroduplexes appeared as intense bands (circled in different color geometric figures of IRD 700 channel and corresponding same color ones of IRD 800 channel). (b) ORG-EcoTILLING gel image of validation in polymorphism site 474 of *accD* gene (Figure S1, Table 7) by using one of the three samples of A64, A65 and A67 as reference with the others. In the channel of IRD700 or IRD800, the lane of 1, 2, and 3; 4, 5 and 6; 7, 8 and 9 were sample A64, A65 and A67, respectively, but the reference of 1, 2, and 3 was A64; the reference of 4, 5 and 6 was A65, and the reference of 7, 8 and 9 was A67. It was obvious that there was different polymorphism in the same position 474 because polymorphism appeared when 65 mixed with A67 (blue arrow showed polymorphism band).

Since this study involved quite distantly related accessions, it was necessary to verify whether the same polymorphic position detected by ORG-EcoTILLING represented identical transversion events within these samples. We therefore designed an experiment to validate the difference for the same polymorphic position discovered by ORG-EcoTILLING among species or higher taxa. Based on the results of ORG-EcoTILLING in the *matK*-2 fragment of *Brassica* species in Figure 3, we selected B76 mutated sample as the reference to mix with B26 (as the reference sample in Figure 3) and 19 mutated samples (also including B76, Figure 3), respectively. The result showed that there was no intense band in samples of B51, B64, B65, B66, B68, B69, B73, B74, B76, B79, B81, B91, B92 and B93, because these mutated samples had the same polymorphisms and positions as the reference sample B76. However, three polymorphic positions were discovered in the lane of B26, identical to those of the reference sample B76 (Figure 3). Interestingly, the lanes for B60, B90, B94, B95 and B96 all showed one polymorphic position (Figure 7a), primarily due to the fact that these samples held the special polymorphism which the reference sample B76 did not share with (Figure 3). Thus, the result clearly demonstrated that the same polymorphic position detected by ORG-EcoTILLING revealed the same polymorphism between *Brassica* intraspecies.

Plants from different genera or tribe shared polymorphisms in *accD* and/or *atp6* whereas they did not share with other accessions of the same species. For example, it was obviously not the case for position 474 in *accD* between samples A65 and A67 (Figure S2). Therefore, we designed an ORG-EcoTILLING experiment to test them by using one of the three samples of A64, A65 and A67 as reference with the others. The result revealed the presence of polymorphisms when A65 was mixed with A67, suggesting that distinct polymorphisms were located at the same position (Figure 7b).

### Phylogenetic relationships of chloroplast genes among three *Brassica* species

**Figure 8:**
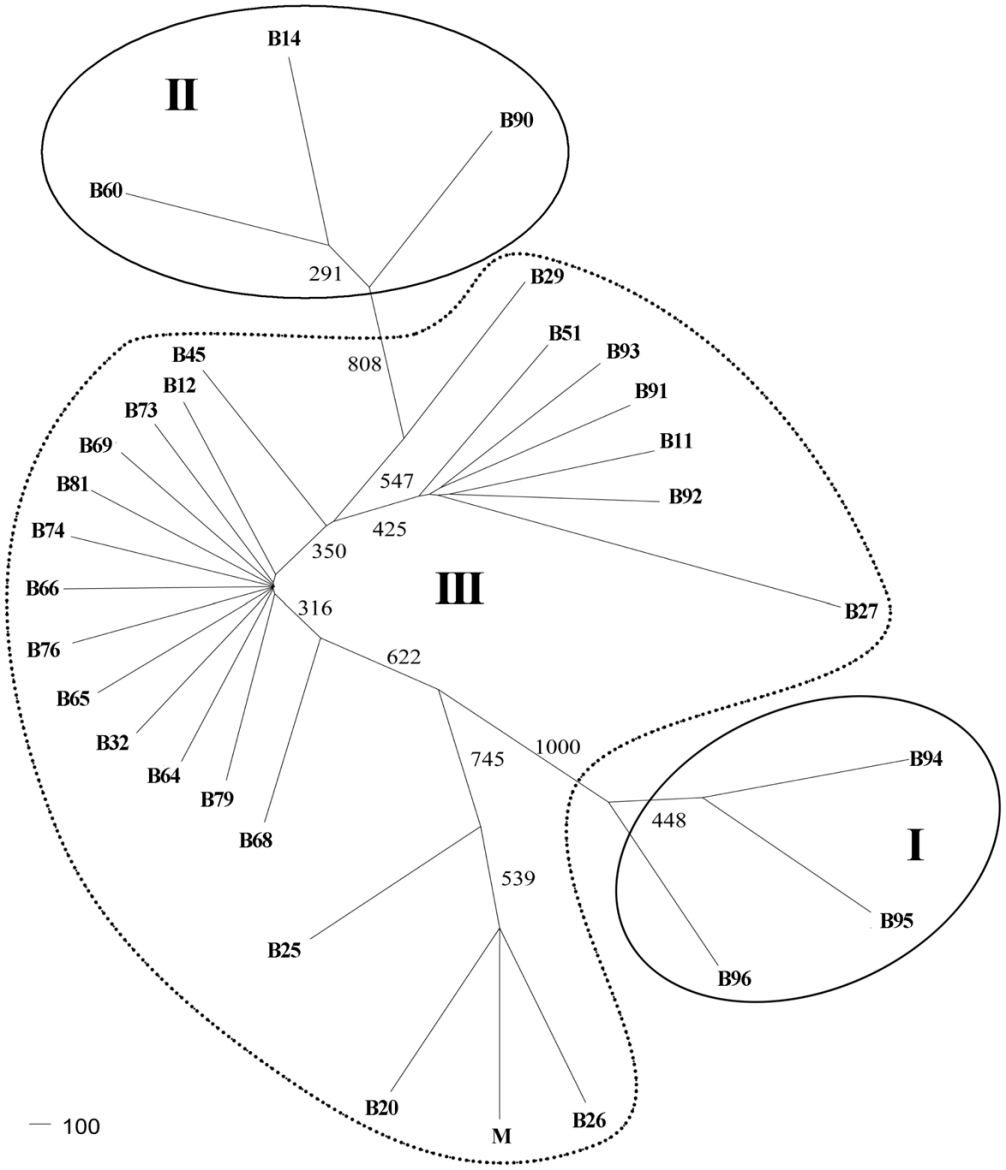
Phylogenetic tree was constructed based on the integrating data of the three chloroplast genes (*accD*, *matK* and *rbcL*) in *Brassica* species. A total of *accD*, *matK* and *rbcL* sequences of 90 accessions of *B. napus* and each of 3 accessions of *B. rapa* and *B. oleracea* were collected for analysis. The tree was constructed by the neighbour-joining method and bootstrap values above 750 from 1000 resamplings were shown for each node. The code in the tree corresponded to the locality code of Table 2. M represented 69 no mutation samples in *B. napus*. The bar corresponded with 100 substitutions per site.

In order to exploit the phylogenetic relationship among the different *Brassica* species based on the polymorphism results by ORG-EcoTILLING, a phylogenetic tree was constructed using 288 chloroplast genes sequences. Ninety DNA sequences representing each of *accD*, *matK* and *rbcL* in *B. napus*, and three sequences of each of three chloroplast genes in *B. oleracea* and *B. rapa* were analysed. Based on the results no mutation was found to have an identical DNA sequence as that of the reference sample, and this greatly reduced the samples available for DNA sequencing. For example, 27 samples were sequenced for *accD* and *matK* (including the reference), and 20 samples for *rbcL* (including the reference). Therefore, it was possible for us to analyze DNA polymorphisms in a large number of samples, merely through the strategy of combining ORG-EcoTILLING results with existing sequences data. The results revealed three distinct classes (I, II and III) within the genus (Figure 8). The three *B. oleracea* accessions were distinctly diverged from both *B. napus* and *B. rapa* accessions, and the majority of *B. napus* clustered with three accessions of *B. rapa*. However, the three accessions of *B. napus*, B14, B60 and B90 clustered in a clade (II), distinct from other accessions.

In conclusion, our results demonstrate that there are obvious differences in the detection rate of cytoplasmic DNA polymorphisms by ORG-EcoTILLING within the Brassicaceae family. At *Brassica* interspecific and intraspecific levels, 100% of mutations were detected in SNPs and INDELs in the *accD*, *matK* and *rbcL* genes. However, this method could not detect all mutation events in the *accD* gene at the genus level in the taxa investigated. In contrast, the detection rate within the *atp6* gene was 100% accurate among the Brassicaceae family. Moreover, the information from ORG-EcoTILLING revealed genetic variations within the species of *B. napus*.

## Discussion

We have demonstrated that a CEL I-based heteroduplex cleavage strategy, which was originally developed for TILLING and EcoTILLING [48], [49], [63], can be successfully applied to the discovery of chloroplast and mitochondrial DNA polymorphisms, especially at the inter- and intraspecific levels.

TILLING and EcoTILLING have been proven to be inexpensive and efficient technologies for the discovery of DNA polymorphisms within specific genes. By applying these methods, much progress has been achieved. However, all existing studies have focused on detecting genetic variation within nuclear genomes of different eukaryotic organisms [44]–[47], [64]–[67]. The results of the present study suggested that the development of ORG-EcoTILLING will provide a high-throughput and cost-effective alternative to the current state-of-the-art techniques for studying chloroplast and mitochondrial genomes. Application of new methods in phylogenetic and ecological studies usually leads to important progress in a range of fields. For example, using chloroplast microsatellites, much progress has been achieved in recent decades in investigations of population genetics, crop plant evolution and domestication and phylogenetics [14]. As ORG-EcoTILLING has several important advantages over chloroplast microsatellites, we can expect widespread application of this new tool in chloroplast and mitochondrial genome studies, with more informative outcomes.

Compared with existing techniques, this method requires only a minimal amount of plant tissue or DNA and avoids the laborious experimental procedures associated with cpDNA and mtDNA purification, DNA enzymatic digestion and Southern hybridization. The use of genomic DNA is one of the key advantages of the successful application of chloroplast microsatellites. We can apply this method to survey natural variation in a region of interest accurately and affordably, which is extremely important for chloroplast and mitochondrial genome research because these genomes share the common characteristic of slow nucleotide substitution rates. If more regions are assessed, the information content will rise accordingly. Point mutations are unevenly distributed in plant chloroplast genomes. Magee *et al.* (2010) found a region of chloroplast DNA in plants related to the sweet pea (*Lathyrus*) in which the local point mutation rate is at least 20 times higher than elsewhere in the same molecule [68]. In this study, it was found that the *accD*, *matK* and *rbcL* genes present different point mutation rates among different regions in *Brassica* species (Table 7), which suggests that more regions need to be studied. ORG-EcoTILLING can efficiently detect rare SNPs and INDELs in specific chloroplast genes with high throughput (including coding regions and non-coding regions). Using a Li-COR gel analyzer, 96 samples can be screened in a single gel run using 1∶1 reference and query pooled samples, and the efficiency is much higher than that of DNA sequencing.

Under the experimental conditions used here, the endonuclease CEL I cuts with partial efficiency, allowing the detection of multiple mismatches in a DNA duplex [48], [69]. We identified both SNPs and small INDELs in chloroplast and mitochondrial gene fragments over a roughly 800-bp window (1 kb-2×100 bp, the terminal noisy regions), similar to EcoTILLING for nuclear genes [48]. However, the degree of detection accuracy varies at different taxon levels. For example, at inter- and intraspecific levels, 10, six and one mutation sites were discovered by ORG-EcoTILLING in the *accD*, *matK* and *rbcL* genes, respectively, and the detection rates of these chloroplast genes were all 100% (Table 6), which corresponded with the results of DNA sequencing. Furthermore, at higher taxonomic levels (among tribes and genera in Brassicaceae), the total detection rate of the chloroplast gene *accD* was only 64%, whereas it was 100% in the mitochondrial gene *atp6* (Table 7).

We found that as the number of mutations detected per fragment increases, the scoring and tracking of cleaved fragments becomes more difficult. The lower accuracy at high taxonomic levels could be attributed to higher numbers of polymorphisms occurring among distant samples at intertribal or intergeneric levels, as in the case of the *accD* gene, from nucleotide 354 to 380 of the *accD* gene in A65, A67, A68 and 69, there were 14 polymorphism sites within a range of 27 bases, but only two of these were detected compared with the results of DNA sequencing (Table 7). Therefore, overestimating the number of mismatches decreased the resolution and signal intensity in the CEL I analysis, which was also found in the case of the PIF2-2 haplotype [48]. Moreover, the accuracy of this method may be reduced in extreme situations such as where multiple mutations occur in a very short region. In this study, only one of 13 polymorphic sites in the *accD* gene was discovered within the first 80 bp (Table 7).

Recently, increasingly complete chloroplast and mitochondrial genome sequences of many species of animals, plants and microbes have been obtained by applying next-generation DNA sequencing technologies [56], [70]–[72]. These complete genome sequences provide important references, but they are still not sufficient for conducting phylogenetic analysis and functional studies of chloroplast and mitochondrial genes, in which more information for individuals in populations is needed. The increased number of polymorphisms screened in organelle DNA is particularly advantageous for evolutionary and ecological studies because the level of DNA polymorphism at inter- and intraspecific levels is generally very low, and our results indicate that ORG-EcoTILLING is ideally suited for the high-throughput, accurate discovery of rare chloroplast and mitochondrial DNA polymorphisms. Therefore, ORG- EcoTILLING is a good alternative for assessment of organelle genome variation.

The most important and attractive application of this method is to conduct genome-wide assessments of chloroplast and mitochondrial DNA diversity within a population at the inter- and intraspecific level, which is particularly important for providing a better understanding of the evolution of organelle genomes and the evolution and ecology of species of interest. By using a gene overlapping strategy, we will be able to construct haplotype maps of chloroplast and mitochondrial genomes efficiently. At present, only a few haplotype maps of the nuclear genomes of humans, Arabidopsis and maize have been constructed [73]–[75]. Unlike the nuclear genome, because the polymorphism level of organelle genomes are much lower, and the recombination is much rarer, it is unnecessary to sequence genome of all individuals of a species for the purpose of haplotype map construction. A more efficient way to do this is to use the ORG-EcoTILLING strategy. The size of the chloroplast genome in plants is 120–200 kb [11]. A single EcoTILLING run generally covers a 1-kb target region; considering overlap and terminal noisy regions. With approximately 200 runs, we can expect to obtain genome-wide coverage of 96 samples and observe the full spectrum of variation. Organelle haplotype maps will provide global documented information on inter- and intraspecific variations, which will greatly facilitate studies of the evolution, domestication, population genetics and gene structure and organization.

The data from ORG-EcoTILLING could be applied to analyze phylogenetic relationships. Our study demonstrated that the three *B. oleracea* accessions were distinctly diverged from both *B. napus* and *B. rapa* accessions, while the majority of *B. napus* clustered with three accessions of *B. rapa*. The probable reason is that most accessions of *B. napus* originated from the cytoplasm of *B. rapa*, suggesting that *B. rapa* was a much more likely maternal progenitor for *B. napus* than *B. oleracea*, which was consistent with previous reports [76], [77]. Moreover, genetic variations were also observed within the *B. napus*, such as B14, B60 and B90, which were diverged from other accessions (Figure 8), most likely due to the multiple origins or evolution in the relatively recent domestication and modern breeding *B. napus*.

Although this study focused on plant chloroplast and mitochondrial DNA polymorphisms, the method developed here is also likely to be applicable to human and animal mitochondrial genomes. The high-throughput 454 method was recently used to sequence the complete human mtDNA genome of 109 individuals from the Philippines, and ∼55-fold coverage was achieved, generating <1% missing data per sequence [78]. If ORG-EcoTILLING is proven to be applicable to human and animal mitochondrial genomes, as the size of human and animal mitochondrial genomes is approximately 16 kb [79], additional genome-wide investigations of mitochondrial genomes will become practical not only in humans, but also in many animal species.

There are some limitations to ORG-EcoTILLING. For example, the method does not directly generate sequence information about the detected mutations, and one representative sample among many individuals sharing identical DNA haplotypes must be sequenced to acquire sequence information for the subset. In this case, complementary DNA sequencing would be needed for the extraordinary region.

The rapid development of NGSts promises the possibility of global studies of chloroplast and mitochondrial genomes at population levels. However, ORG- EcoTILLING is ideally suited to screening large numbers to survey populations and diversity collections in chloroplast and mitochondrial genomes, thus reducing samples for which sequencing would provide more detailed information. Therefore, ORG-EcoTILLING represents an alternative new tool for high-throughput, high-resolution analysis of organelle genomes.

## Supporting Information

Figure S1 **A schematic representation of the position of the primers on** ***accD*** **,** ***matK*** **,** ***rbcL*** **and atp6 genes.** Two pairs of primers were designed in *accD*, *matK* and *rbcL* of 96 *Brassica* species, whereas only one pair of labeled primer was designed in *accD* and *atp6* genes of 91 accessions from Cruciferae. Black boxes showed the genes, and detailed information on each primer was shown in Table 3.
(TIF)

Figure S2 **Alignment of representative DNA sequences showing polymorphism in** ***accD*** **gene region regarding INDELs and substitutions.** The sequence in each row is a representative sequence, and the red line showed the sequence of the reference sample A64. The numbers in parentheses show the number of samples in Brassicaceae. Blue arrows above the alignment indicated the position of mutation. The position showed in the sequence map began from initial primers attached to M13 adaptors.
(TIF)

Figure S3 **Alignment of representative DNA sequences showing polymorphism in** ***atp6*** **gene region regarding INDELs and substitutions.** The sequence in each row is a representative sequence, and the red line showed the sequence of the reference sample A24. The numbers in parentheses show the number of samples in Brassicaceae. Blue arrows above the alignment indicated the position of mutation. The result of DNA sequencing was DNA antisense strand of *atp6* gene.
(TIF)

Table S1 **Analysis of ORG-EcoTILLING information in 700 and 800 channel from** ***accD*** **,** ***matK*** **and** ***rbcL*** **gene.**
(XLS)

Table S2 **Band analysis of** ***accD*** **gene in 91 Brassicaceae samples identified by ORG-EcoTILLING.**
(XLS)
