# Supplementary figures and images for: High-Throughput Discovery of Chloroplast and Mitochondrial DNA Polymorphisms in Brassicaceae Species by ORG-EcoTILLING
Source: PLoS One. 2012 Nov 21;7(11):e47284. doi: 10.1371/journal.pone.0047284 (PMC3504036; doi:10.1371/journal.pone.0047284)

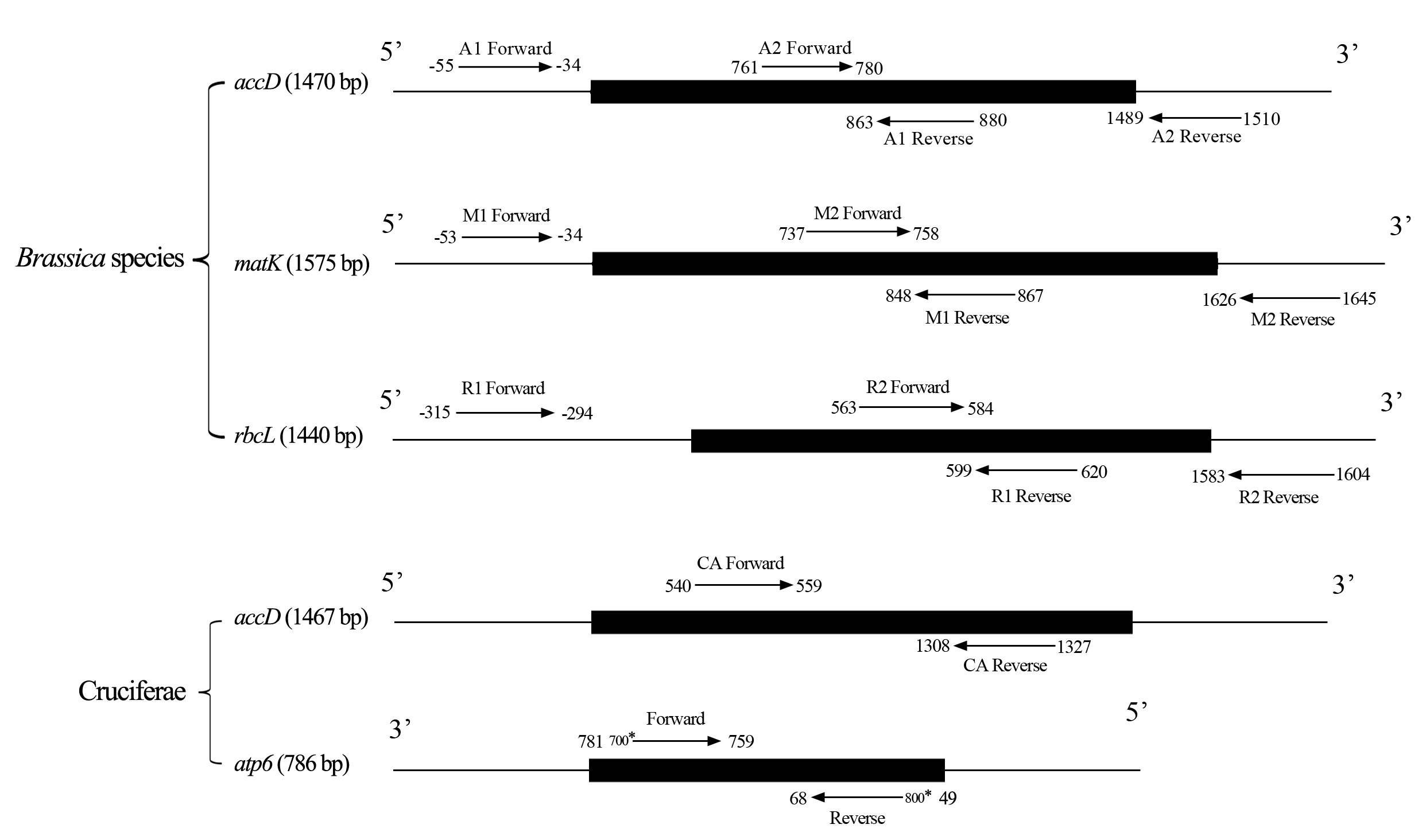

Supplement: Figure S1 — A schematic representation of the position of the primers on accD , matK , rbcL and atp6 genes. Two pairs of primers were designed in accD, matK and rbcL of 96 Brassica species, whereas only one pair of labeled primer was designed in accD and atp6 genes of 91 accessions from Cruciferae. Black boxes showed the genes, and detailed information on each primer was shown in Table 3. (TIF) [file pone.0047284.s001.tif]

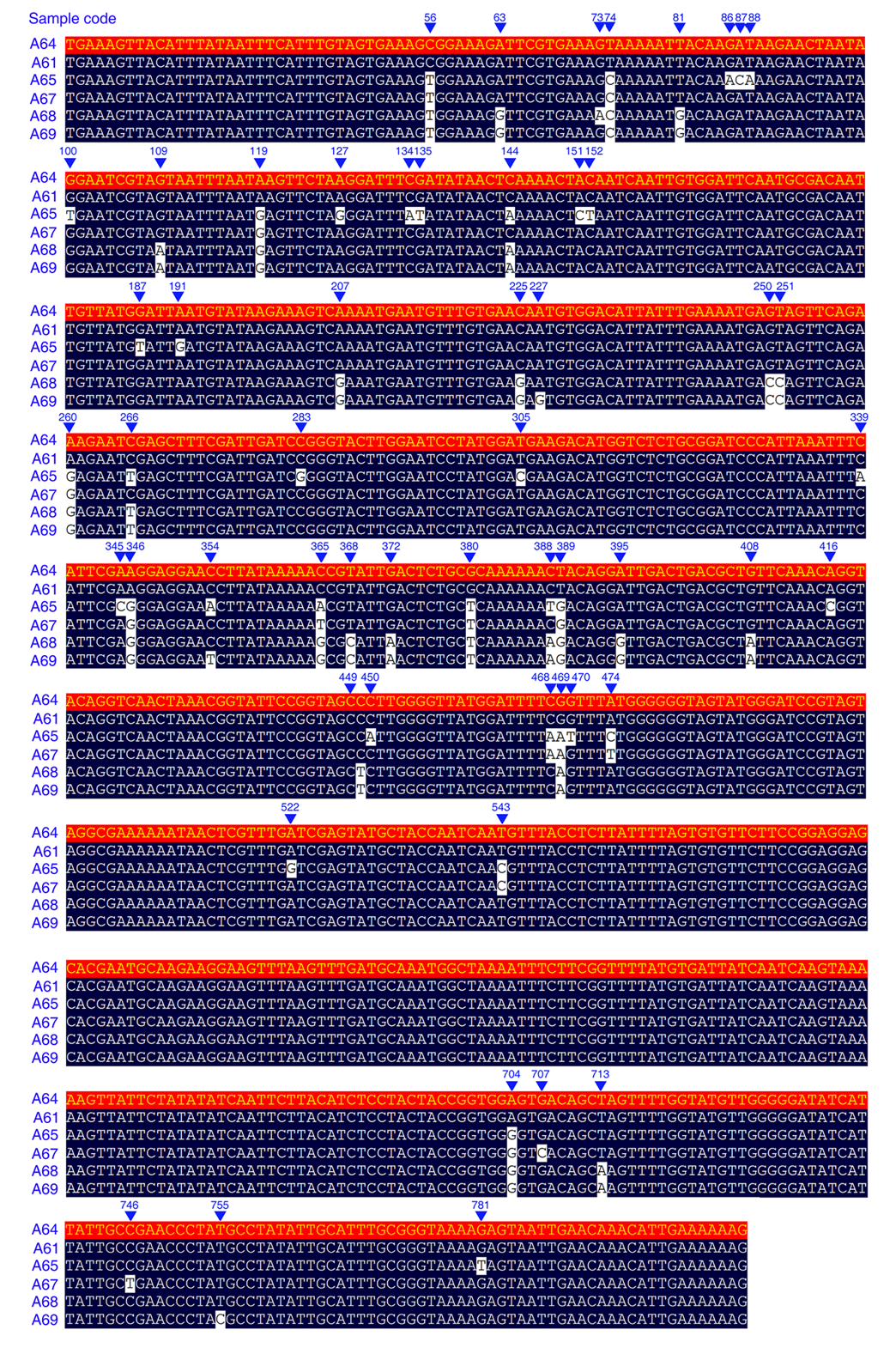

Supplement: Figure S2 — Alignment of representative DNA sequences showing polymorphism in accD gene region regarding INDELs and substitutions. The sequence in each row is a representative sequence, and the red line showed the sequence of the reference sample A64. The numbers in parentheses show the number of samples in Brassicaceae. Blue arrows above the alignment indicated the position of mutation. The position showed in the sequence map began from initial primers attached to M13 adaptors. (TIF) [file pone.0047284.s002.tif]

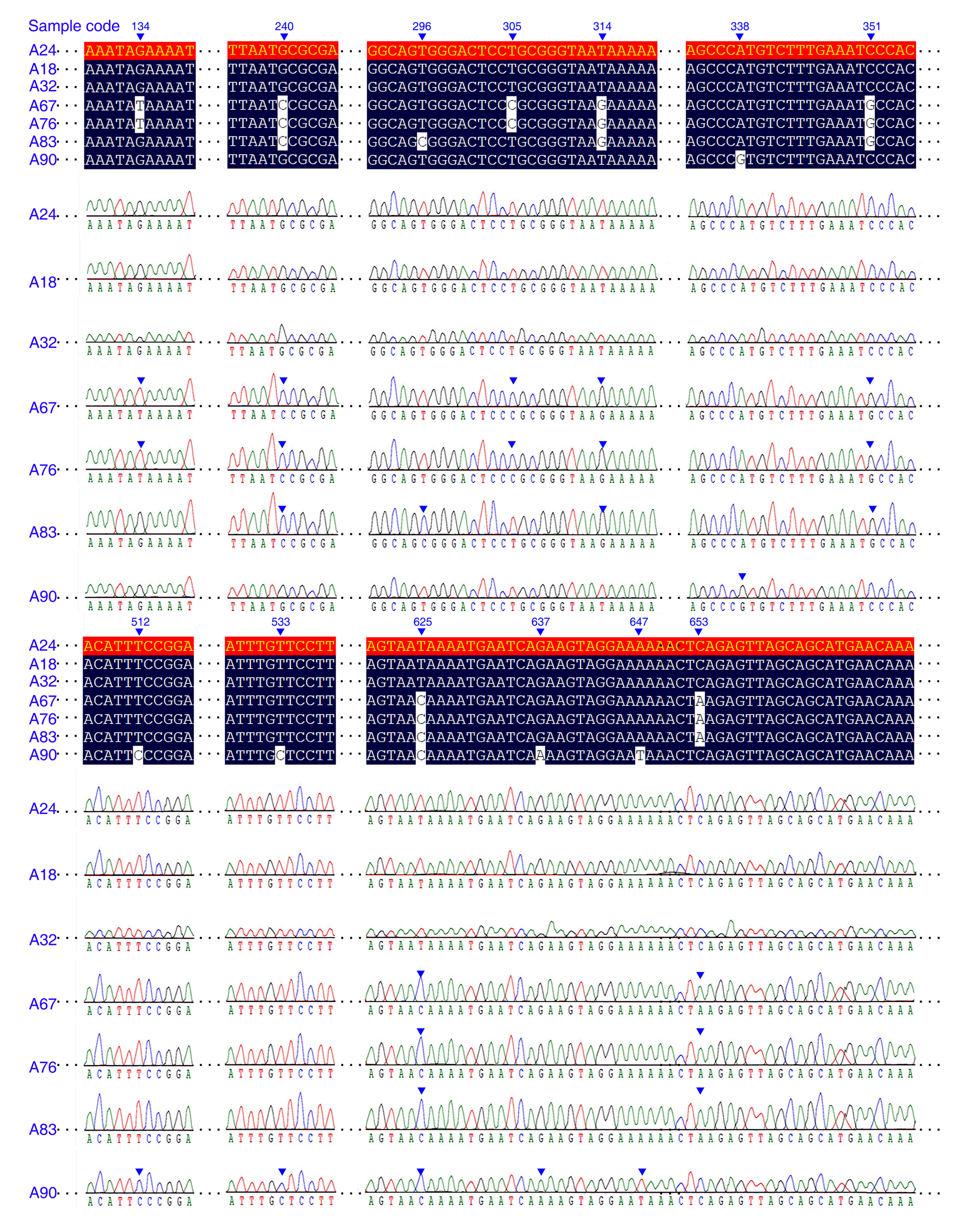

Supplement: Figure S3 — Alignment of representative DNA sequences showing polymorphism in atp6 gene region regarding INDELs and substitutions. The sequence in each row is a representative sequence, and the red line showed the sequence of the reference sample A24. The numbers in parentheses show the number of samples in Brassicaceae. Blue arrows above the alignment indicated the position of mutation. The result of DNA sequencing was DNA antisense strand of atp6 gene. (TIF) [file pone.0047284.s003.tif]
